# Single-Cell Based Quantitative Assay of Chromosome Transmission Fidelity

**DOI:** 10.1534/g3.115.017913

**Published:** 2015-03-30

**Authors:** Jin Zhu, Dominic Heinecke, Wahid A. Mulla, William D. Bradford, Boris Rubinstein, Andrew Box, Jeffrey S. Haug, Rong Li

**Affiliations:** * Stowers Institute for Medical Research, 1000 East 50th Street, Kansas City, Missouri 64110; † Department of Molecular and Integrative Physiology, University of Kansas Medical Center, 3901 Rainbow Boulevard, Kansas City, Kansas 66160

**Keywords:** mitosis, chromosome instability, qCTF assay, dosage-sensitive gene

## Abstract

Errors in mitosis are a primary cause of chromosome instability (CIN), generating aneuploid progeny cells. Whereas a variety of factors can influence CIN, under most conditions mitotic errors are rare events that have been difficult to measure accurately. Here we report a green fluorescent protein−based quantitative chromosome transmission fidelity (qCTF) assay in budding yeast that allows sensitive and quantitative detection of CIN and can be easily adapted to high-throughput analysis. Using the qCTF assay, we performed genome-wide quantitative profiling of genes that affect CIN in a dosage-dependent manner and identified genes that elevate CIN when either increased (icCIN) or decreased in copy number (dcCIN). Unexpectedly, qCTF screening also revealed genes whose change in copy number quantitatively suppress CIN, suggesting that the basal error rate of the wild-type genome is not minimized, but rather, may have evolved toward an optimal level that balances both stability and low-level karyotype variation for evolutionary adaptation.

A fundamental requirement for mitotic proliferation of eukaryotic cells is to accurately transmit chromosomes such that individual cells of the population inherit identical numbers of each chromosome. In the context of this study, we refer to chromosome instability (CIN) as a measure of the likelihood of mitotic errors occurring in a euploid cell population that produces aneuploid progeny cells exhibiting gain or loss of chromosomes. A broader definition of CIN also includes segmental or structural changes of chromosomes (Geigl *et al.* 2008). Recent studies have shown that aneuploidy, due to imbalanced chromosome stoichiometry, alters the relative expression level of many genes and can lead to dramatically modified cellular phenotypes (Henry *et al.* 2010; Pavelka *et al.* 2010; Torres *et al.* 2007). Thus, to maintain genotypic and phenotypic stability, euploid organisms have evolved intricate mechanisms to ensure mitotic fidelity and suppress CIN (Musacchio and Salmon 2007). In euploid unicellular organisms, including the budding yeast *Saccharomyces cerevisiae*, CIN was estimated to be exceedingly low, with a mitotic error rate on the order of 1 in 10^6^ cell divisions (Kumaran *et al.* 2013). In most somatic cell types of metazoans, CIN is also expected to be low, although direct measurements have been more difficult. The majority of solid tumor cells, on the other hand, exhibit moderate to drastically elevated CIN and other chromosome abnormalities (McGranahan *et al.* 2012). High CIN in tumors often predicts poor prognosis, and there is increasing evidence pointing to aneuploidy as an important form of driver mutation during tumor evolution (Davoli *et al.* 2013; Potapova *et al.* 2013).

Understanding the mechanisms that maintain genome stability in normal cells or cause elevated CIN in cancer necessitates the development of reliable and highly quantitative methods for measuring CIN. During the past two decades, several assays in budding yeast that use different principles were developed to measure CIN on a semiquantitative level, enabling fruitful studies of genes and mechanisms that confer accurate chromosome segregation in this model organism. These existing CIN assays are based on changes in growth ability or color of yeast colonies triggered by the loss of certain native or artificial chromosomes (Stirling *et al.* 2011). The chromosome transmission fidelity (CTF) assay measures whole chromosome loss by using an artificial chromosome. The a-like faker (ALF) assay uses a readout that can be the consequence of a whole chromosome loss, chromosome rearrangements, or gene conversion. Finally, the gross-chromosomal rearrangements assay only detects terminal chromosomal deletions (Chen and Kolodner 1999; Spencer *et al.* 1990; Yuen *et al.* 2007). These assays in combination with genome-wide open reading frame (ORF) deletion or conditional mutant libraries led to the identification of 692 CIN genes in yeast (Stirling *et al.* 2011). Despite their usefulness, these assays lack quantitative rigor as the result of two main factors. First, the readouts of these assays are several steps downstream of the actual CIN event and may be complicated by defects or noise in intermediary processes such as mating, colony growth, and/or colony color development. Second, even where CIN has been elevated by tens to hundreds of fold, mitotic errors are stochastic rare events that require large population size for accurate rate measurement. However, examining the use of existing assays to score thousands to millions of colonies can be both costly and labor-intensive. The combined effects of these two factors make existing assays semiquantitative at best and prone to fluctuations and false readouts, especially when adapted to genome-scale analysis.

In this study, we developed a single cell−based, quantitative chromosome transmission fidelity (qCTF) assay for the measurement of chromosome transmission fidelity in yeast that can be performed in either low- or high-throughput formats and is based on a principle that can be extended to metazoans and other multicellular organisms. The high precision and reduced labor costs of qCTF allowed us to carry out two genome-wide screens examining quantitative effects of gene copy number changes on CIN that we report here. Our results validate the qCTF assay in yeast and demonstrate its utility in studying the genetics of processes regulating CIN in eukaryotes.

## Methods and Materials

Strain and plasmid information can be found in Supporting Information, Table S1. Polymerase chain reaction (PCR) and quantitative polymerase chain reaction (qPCR) primers can be found in Table S2.

### Construction of the yeast reporter strains RLY8492 and RLY8493

The MATα locus was inserted into the pRS305 vector, containing a *LEU2* marker. Together the *MATα* locus and *LEU2* marker sequence were amplified using PCR and transformed into YPH278 to replace the *SUP11* and *URA3* sequence on the present mini-chromosome (MC). The resulting strain was then mated with the RLY2626 strain, which is modified to have the a-specific *MFA1* gene tagged with 3×GFP::HIS5, resulting a strong fluorescent reporter. After inducing sporulation, the meiotic progeny were screened for His^+^, Leu^+^, and *ADE2* growth phenotype, resulting in the desired RLY8492 strain. To generate the RLY8493 strain, the *MATa* locus of the RLY8492 strain was deleted using the *natMX* module contained in the pFA6a-natMX plasmid.

### Construction of MoBY-ORF control plasmid RLB884

The MoBY-ORF control plasmid pJZ013 was constructed as described previously (Ho *et al.* 2009). In short, the KanMX4 module was amplified with PCR from the pFA6a-KanMX4 plasmid and subsequently cotransformed into the RLY2626 S288c wild-type (WT) strain together with the *XhoI*-linearized p5472 plasmid as backbone (Ho *et al.* 2009). Yeast colonies growing on SC-Ura+G418 media plates were used to inoculate SC-Ura+G418 liquid medium from which the plasmid was recovered using a Zymoprep Yeast Plasmid Miniprep II Kit (D2004; Zymo Research, Irvine, CA). The isolated plasmid was then transformed into the bacterial host strain BUN20 (Li and Elledge 2005), resulting in the bacterial strain RLB884. For confirmation the RLB884 plasmid was isolated from the bacterial RLB884 strain using the GenEluteTM Plasmid Miniprep kit (PLN350-1KT; Sigma Life Science) and subsequently sequenced to confirm the presence of the UPTAG- (TATTTACGCGGGA-GACTCGT) and DOWNTAG- (ATACACGTCGAAGGAGTGCC) barcode.

### Construction of strains for dcCIN gene screen

RLY8493 cells were pinned in quadruplicates from 96-well plate glycerol stocks onto SC-Leu + CloNat (100 µg/mL) agar plates using the Singer RoToR HDA Robot (RO7026-100Y; Singer Instruments) with 96 long Pin Repads (RP-MP-2L; Singer Scientific). The MATa haploid yeast Knockout (KO) collection was pinned from 384-well plate glycerol stocks onto YPD with G418 (200 µg/mL) agar plates using the Singer RoToR HDA Robot with 384 long Pin Repads (RP-MP-3L; Singer Scientific). Strains of both mating type were then grown for 2−3 d at 30° in a low-temperature 815 Precision Incubator (3721; Thermo Electronic Corporation). When the colonies reached a sufficient size, RLY8493 cells and MATa haploid KO cells were mixed on YPD agar plates using the Singer RoToR HDA Robot with 384 short Pin Repads (RP-MP-384; Singer Scientific). The cells from the RLY8493 strain were pinned onto the YPD plates first, followed by the cells of MATa KO strain on top. The cells were then incubated at 30° overnight (O/N) in an 815 Precision Incubator. After incubation, diploids were selected-for by pinning from the YPD+ plates onto SC-Leu + CloNat (100 µg/mL) + G418 (200 µg/mL) agar plates using the Singer RoToR HDA Robot with 384 short Pin Repads. The cells were then incubated at 30° for 1−2 d. After incubation, the cells were transferred into an untreated 96-well flat bottom microplate with lid (222-8030-F1K; Evergreen Scientific) containing SC-Leu + CloNat (100 µg/mL) + G418 (200 µg/mL) liquid medium using the Singer RoToR HDA Robot with 96 long Pin Repads. The 96-well microtiter plates were sealed with breathable Airpore Tape Sheets (19571; QIAGEN) and allowed to grow for 1−2 d at 30°/220 rpm in a Multitron Infors Shaking Incubator (80120805BC2; ATR Biotech). From the resulting cell suspension 100 µL were transferred into 100 µL of 50% Glycerol (1:2 dilution) in each well of a new 96-well microtiter plate. Prepared untreated 96-well flat bottom microplates with lids were then sealed using Seal & Sample Aluminum Foil Lids (536619; Beckman Coulter) and stored until use in a Revco Ultima II −80° Freezer (Ult2586-9-A38; Kendro Laboratory Products).

### Construction of strains for the icCIN gene screen

The MoBY-ORF library collection was transformed in a high-throughput manner into the previously constructed RLY8492 yeast strain in a 96-well format using the BioMek FX Laboratory Automation Workstation/Biomek Software (717013; Beckman Coulter). To summarize, RLY8492 strain was inoculated into 200 mL of YPD liquid medium and incubated O/N at 30°/250 rpm in a Multitron Infors Shaking Incubator (80120805BC2; ATR Biotech). After ∼12 hr OD_600_ was measured using an Ultrospec 3100 pro spectrophotometer (80-2112-31; Amersham Biosciences) and diluted to an OD_600_ of 0.1 using fresh YPD liquid medium. The cell culture was further incubated at 30°/250 rpm for 3 hr after which the cell culture was transferred into 50-mL centrifuge tubes with screw caps (21008-178; VWR) and spun down at 1734 g for 3 min using an Allegra X-22R centrifuge. Supernatant was aspirated, and the pellet was washed twice with 5 mL of double-distilled water (ddH_2_O), once with 20 mL of 0.1 M LiOAc, and then resuspended in 1.25 mL of 0.1 M LiOAc. The prepared yeast cell suspension was then added to 1.875 mL of 1 M LiOAc, 0.625 mL of ddH_2_O, 2.5 mL of 2 mg/mL denatured/sheared salmon sperm DNA, and 12.5 mL of 50% polyethylene glycol 3350. Then, 100 µL of yeast transformation mix was distributed into each well of a 96-well half skirt PCR plate (MPX-96M2; Phenix Research Products), after which 10 µL of MoBY-ORF plasmid was added and repeatedly mixed using the BioMek FX Laboratory Automation Workstation/Biomek Software. The 96-well half skirt PCR plate containing the transformation mix was then covered with breathable Airpore Tape Sheets (19571; QIAGEN) and heat-shocked at 42°/220 rpm for 1 hr using a Multitron Infors Incubator. After heat shock the PCR plate was spun down at 1180 g for 5 min using an Allegra 25R Centrifuge (425752; Beckman Coulter) and the supernatant was aspirated. Pellets were resuspended in 15 µL of ddH_2_O, resealed with breathable Airpore Tape Sheets and shaken for 5 min at 1500 rpm using a Eppendorf Mixmate (PCB-11; Eppendorf). From the resuspended wells, 7 µL were spotted onto SC-Ura agar plates. SC-Ura agar plates were grown at 30° in a low-temperature 815 Precision Incubator (3721; Thermo Electronic Corporation) for 1 d. Transformants were picked with a 96 Solid Pin Multi-Blot Replicator (VP407; V&P Scientific) and used to inoculate 100 µL of SC-Ura liquid medium in an untreated 96-well flat bottom microplate with lid (222-8030-F1K; Evergreen Scientific). The cell culture was incubated O/N at 30°/220 rpm using a Multitron Infors Incubator and then mixed with 100 µL of 50% glycerol to make a saturated library stock. Prepared untreated 96-well flat bottom microplates with lids were then sealed using seal & sample aluminum foil lids (536619; Beckman Coulter) and stored until use in a Revco Ultima II −80° Freezer (Ult2586-9-A38; Kendro Laboratory Products).

### High-throughput CIN screening procedure in yeast

For the dcCIN screen, 30 µL of glycerol stock was used to inoculate 1.5 mL of SC-Leu medium (1:50 dilution) in a 2.2-mL deep-well plate, sterile pp wells w/conical bottom (M1810S; PHENIX Research Products) using a Biomek FX Laboratory Automation Workstation/Biomek Software (717013; Beckman Coulter). Subsequently the 2.2-mL Deep Well Plate containing the cell suspension was sealed with AeraSeal Sterile Microporous Sealing Film (LMT-AERAS-EX; PHENIX Research Products) and incubated for 24 hr, attached to a TC-7 Rotor (500484410; New Brunswick Scientific) set to speed level 10, inside a low temperature 815 Precision Incubator (3721; Thermo Electronic Corporation) at 30°. After the first growth phase, the block was mixed for 5 min using an Eppendorf Mixmate (PCB-11; Eppendorf) set to 1400 rpm and 23 µL of cell suspension was diluted in 207 µL of SC-Leu medium inside a 96-well nontissue culture plate with flat-bottom and low-evaporation Lid (351172; BD Falcon). The 96-well plate was shaken for 3 min at 1000 rpm using an Eppendorf Mixmate and the OD_600_ of each strain was measured on a SpectraMax M2 Multimode Microplate reader (DE05224; Molecular Devices). After this step, 30 µL of the cell suspension was diluted into 1.5 mL of SC-Complete medium (1:50 dilution) in a 2.2-ml deep well plate using the Biomek FX Laboratory Automation Workstation. The remaining 200 µL of cell suspension inside the 96-well plate were spun down using an Allegra 25R Centrifuge (425752; Beckman Coulter) set to 3000 rpm for 3 min and the supernatant aspirated by the Biomek FX Laboratory Automation Workstation.

The cell pellets were resuspended in 200 µL of 4% paraformaldehyde , shaken for 3 min at 1000 rpm using a Eppendorf Mixmate, and fixed for 15 min at room temperature (RT). The fixed cells were then washed twice by centrifugation using the Allegra 25R Centrifuge set to 3000 rpm for 3 min, the supernatant was aspirated with the Biomek FX Laboratory Automation Workstation, and cell pellets were resuspended in 200 µL of phosphate-buffered saline, pH 7.4. For the last wash, the fixed cells were spun down using the Allegra 25R Centrifuge set to 1698 g for 3 min, resuspended in 250 µL of phosphate-buffered saline, pH 7.4, and shaken on the Eppendorf Mixmate for 3 min at 1000 rpm. The plates containing the fixed and washed cells were then stored at 4° in the dark for flow-cytometry analysis. The new 2.2-mL deep well plate inoculated in SC-Ura was again sealed with AeraSeal Sterile Microporous Sealing Film and incubated for 24 hr attached to a TC-7 Rotor set to speed level 10 inside a low temperature 815 Precision Incubator at 30°. After the second growth phase, the previously described screening process was repeated with the only exception being that no new 2.2-mL deep well plate was inoculated and the current 2.2-mL deep well plate was stored in a Revco Ultima II −80° Freezer (Ult2586-9-A38; Kendro Laboratory Products:) for later confirmation.

For the icCIN screen, the same process was followed except for the fact that the cells were first grown in SD-Ura-Leu liquid medium and then in SC-Ura liquid medium.

### Flow-cytometric analysis of screening samples

Fixed cells contained inside the 96-well nontissue culture plate with flat-bottom and low-evaporation lid (351172; BD Falcon) were transferred into a 96-well MatriTube Storage Plate with clear tubes (MSP096-A; Matrical Bioscience) and covered with a SonicMan Pin Lid (SL0096-P19-SS; Matrical Bioscience). The 96-well MatriTubeTM Storage Plate was then sonicated for 20 sec at 50% power using a SonicMan HT-sonication instrument (SCM1000; Matrical Bioscience). Sonicated cells were then transferred back into an untreated 96-well flat bottom microplate with lid (222-8030-F1K; Evergreen Scientific) and placed on the MACSQuant Analyzer (130-092-197; Miltenyi Biotec) to count the number of green fluorescent protein (GFP)-positive cells in the cell population. For both screens, 125 µL of mixed cell suspension was run per sample. Then, 0.3 million events were counted for each dcCIN screen sample and 0.2 million each for the icCIN samples. Data acquired on the MACSQuant Analyzer were analyzed with FlowJo software 7.6.5 (Tree Star) to calculate the percentage of GFP-positive cells. The previously recorded OD_600_ readings and the differences in frequency of GFP-positive events were entered into Excel2010 (Microsoft Office, version 14.0.7106.5003) to calculate the CIN rate. The loss rate [frequency of cells that lose MCs per generation] was calculated using equation (9) in File S1.

### Isolation and sequence confirmation of MoBY-ORF plasmids

Sterile PP 2.2-mL deep well plates with conical bottoms (M1810S; PHENIX Research Products) were thawed at RT. The complete 1.5 mL contained in the well was transferred to a 2-mL Eppendorf Safe-Lock Tubes (0030 120.094; Eppendorf). Plasmid isolation was performed according to the standard protocol for liquid culture of the Zymoprep Yeast Plasmid Miniprep II kit (D2004; Zymo Research) with two important changes. First, the centrifugation of the culture was performed for 3 min at 13,200 rpm in a Eppendorf Centrifuge 5415D (022621408; Eppendorf) and second, instead of the Zylomylase incubation at 37° the cell pellet was resuspended in Solution 1 was bead beaten for 10 min using 50 µL of 0.6-mm acid-washed glass beads (G8772-10G; Sigma-Aldrich) on a Fisher Vortex Genie 2 (12-812; Fisher Scientific). The ORF region of the isolated plasmid was sequenced using the standard MoBY-ORF primers for the 5′ (ACGTTCAGACGTATCAGTACATCACGAGACTACTA) and 3′ (ATGTTACTTACCACATCACGATAGGTCTCACGATC) position.

### Manual transformations of individual plasmids into *S. cerevisiae*

To transform plasmids into budding yeast, we inoculated 5 mL of YPD liquid medium with a single yeast colony and incubated it O/N at 30°/230 rpm in a Multitron Infors Shaking Incubator (80120805BC2; ATR Biotech). The culture was then diluted up to 50 mL (1:10 dilution) with fresh YPD liquid medium and incubated for 4 hr at 30°/230 rpm. Salmon sperm DNA was boiled for 10 min in boiling water and then put on ice for 5 min. Cells were centrifuged for 3 min/1734 g. The cell pellet was washed with 1 mL of 1M lithium acetate and transferred into a 1.5-mL tube. Cells were centrifuged for 3 min/1734 g. The cell pellet was resuspended in 1 volume of 1 M lithium acetate. Transformation vials were prepared by mixing, (1) 12-μL cell suspension, (2) 5-μL boiled salmon sperm DNA, (3) ∼200- to 500-ng plasmid DNA, and (4) 45 -μL of 50% polyethylene glycol. Samples were incubated for 1 hr at RT, after which 6 μL of 60% glycerol was added followed by 1 hr additional incubation at RT. Cells were heat shocked at 45° for 10 min in a water bath. The cells were then transferred to selective agar plates and incubated at 30° for 24−48 hr.

### Increasing gene copy number by genomic integration with qPCR confirmation

To integrate additional copies of *NPL3* or *MCD1* into the genome, we PCR-amplified the corresponding gene from RLY2626 genomic DNA (gDNA) and ligated it into *XhoI*- or *SacI*-digested pRS306, an integrating plasmid with a *URA3* marker. The resulting construct was transformed into Top10 bacterial cells from which colonies were picked and amplified in 2xYT + Amp liquid medium. Plasmid isolation was performed using the GenElute Plasmid Miniprep kit (PLN350-1KT; Sigma Life Science) followed by sequencing with standard T3 (GCAATTAACCCTCACTAAAGG) and T7 (TAATACGACTCACTATAGGG) primers to determine the presence of the inserted gene. The plasmid was then digested using a restriction enzyme (*AseI* for *NPL3*, *AflII* for *MCD1*), which cuts once inside the middle of the gene sequence and transformed into the RLY8492 yeast strain, containing the MC. The transformed cells were plated on SD-Ura-Leu plates and grown at 30° in a low temperature 815 Precision Incubator (3721; Thermo Electronic Corporation) for 2 d. After incubation, 18 individual colonies were picked and amplified in SD-Ura-Leu liquid medium, from which gDNA was extracted using the standard protocol from the Masterpure Yeast DNA Purification Kit (MPY80200; Epicenter). The qPCR analysis, to determine gene copy number variation, was done in two steps. First, three different primer pairs for the gene of interest were validated for their specificity using haploid WT gDNA and including *ACT1* as endogenous control. Haploid WT gDNA was diluted in series (1:5 dilution) ranging from 100 ng/µL to 0.0064 ng/µL and mixed with 5 µM forward + reverse primer mix inside a Microamp Optical 384-well Reaction plate with barcode (4309849; Applied Biosystems) using a CAS-4200 Liquid Handling System (CAS4200; Corbett Robotics). The prepared Microamp Optical 384-well Reaction plate with barcode was sealed with MicroAmp Optical Adhesive Films (4311971; Applied Biosystems), spun down for 15 sec and analyzed with 7900HT Fast Real-Time PCR system (4329002; Applied Biosystems). Second, the primer pair and gDNA concentration giving the best peak specificity was chosen for the actual copy number determination. gDNA from each of the 18 colonies and one haploid WT was combined with the previously determined most specific primer pair as well as four endogenous controls, namely *ACT1*, *TUB1*, *CDC28*, and *ZWF1*. The Microamp Optical 384-well Reaction plate with barcode, containing 4 replicates for gDNA with each primer combination, was prepared with the CAS-4200 Liquid Handling System. The prepared Microamp Optical 384-well Reaction plate with barcode was sealed with MicroAmp Optical Adhesive Films, spun down for 15 sec and analyzed with 7900HT Fast Real-Time PCR system. The analysis of the gene copy number was done using the Biogazelle qbase PLUS 2.6 system.

### Construction of *NPL3* mutant plasmids, *ESP1* plasmid, *MAD2* plasmid

Two DNA fragments containing promoter, coding region and terminator sequence of *NPL3* point mutant were PCR amplified respectively from plasmid pPS876 (Lee *et al.* 1996). DNA fragments containing *ESP1* or *MAD2* gene were PCR amplified from RLY2626 genomic DNA. Each fragment was then ligated into the *Xho*I-linearized pRS316 (*NPL3*) or pRS314 (*ESP1*, *MAD2*) plasmid with Quick Ligation Kit (M2200S; New England Biolabs).

### Yeast doubling time measurement

Growth curves were obtained from liquid cultures grown in 96-well plates and monitored every 15 min by absorbance at 595 nm using a TECAN Infinite M200 plate reader at 30°. Before the start of the experiment, freshly revived colonies of the indicated strains were grown O/N in selective medium to maintain the MC and plasmids, refreshed in selective medium for 4−5 hr, and then normalized to the starting optical density (OD) of 0.05. The growth rate was defined as a value proportional to the value of the maximal slope of the log-scaled OD curve. The curve was interpolated by splines of the second order, and the first derivative of the interpolated function was found numerically. The maximum of the first derivative was then computed generating an approximate value of the growth rate k, and the doubling time was found as Ln(2)/k. All computations were performed using Mathematica (Wolfram Research, IL).

### Protein interaction network analysis of validated candidate genes

High confidence (0.700) known and predicted protein−protein interaction data were retrieved from STRING v9.1 (Franceschini *et al.* 2013). The interaction network was visualized using Cytoscape 3.1 software (Shannon *et al.* 2003) with a circular layout.

### Gene Onology (GO) analysis of validated candidate genes

dcCIN genes and icCIN genes were classified using GO Slim Mapper tool from SGD (Saccharomyces Genome Database). GO enrichment analysis was performed with WebGestalt(Wang *et al.* 2013) .

### Statistical analysis

All statistical analyses were performed with GraphPad Prism software (GraphPad Software, Inc., San Diego, CA).

## Results

### A fluorescence-based, single-cell assay for quantitative CIN measurement

**Figure 1:**
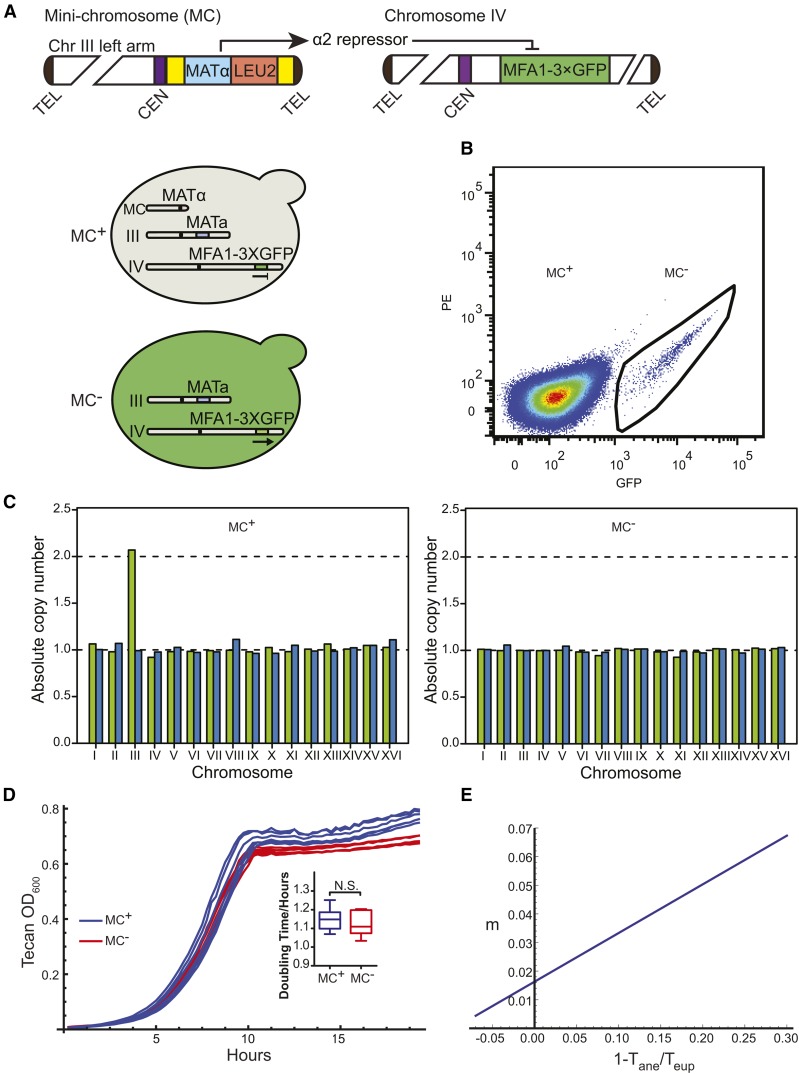
Design and validation of the quantitative chromosome transmission fidelity (qCTF) assay. (A) Illustration of the organization of the mini-chromosome (MC) and how the presence of MC represses the expression of a-specific gene *MFA1*. The yellow bars flanking *MATα* and *LEU2* loci represent pBR322 DNA. TEL, telomere; CEN, centromere. Chromosomes are not drawn proportionally to actual size. (B) Flow cytometry analysis of qCTF strain grown in nonselective media for MC. Black line outlines the small population of highly fluorescence cells that had lost MC. (C) Representative quantitative polymerase chain reaction karyotyping of colonies from GFP^−^ (MC^+^) and GFP^+^ (MC^−^) cells. Roman number indicates each yeast chromosome. The green bar represents the left arm and the blue bar represents right arm. (D) Growth curves of cell with or without MC. Box-and-whisker plots summarize the minimum doubling time of cell with or without MC. The center line of the box indicates the median value, and boxes indicate the interquartile range (IQR). The bottom whisker contains data points that are within 1.5 IQR of the lower quartile and the top whisker contains data points that are within 1.5 IQR of the upper quartile. A *P*-value of 0.503 was calculated from *t*-test with n = 6. N.S., nonsignificant. (E) The linearized dependence of the loss rate of a given chromosome on the ratio of doubling times of cells with normal copy number of this chromosome (eup) and that of cells that have lost a copy of the chromosome (ane). An example was plotted with data in (D) using the equations (13) and (14) in File S1.

The basic principle for our qCTF assay is to provide a direct readout of a chromosome loss event that can be detected in single cells. To this end, we engineered a system that leads to irreversible gain of GFP fluorescence signal soon after the loss of a tester chromosome, which can be detected and quantified in large populations of cells by the use of flow cytometry. Using the yeast mating type-determination system (Haber 2012) we first tagged the *MATa*-specific *MFA1* gene with a 3×GFP fusion at the 3′ end of the ORF by homologous recombination, because Mfa1p is the greatest-expressed *MATa* specific protein (Ghaemmaghami *et al.* 2003). The *MATα* locus was then introduced into the tester chromosome (see below) in a haploid *MATa* strain. The α2 transcriptional repressor produced from the *MATα* locus strongly repressed MATa-specific genes, such as Mfa1p-3×GFP. Thus, when the tester chromosome is present, Mfa1p-3×GFP expression is strongly repressed; however, if this chromosome is lost, Mfa1p-3×GFP will be switched on and the cell will become highly fluorescent within one cell cycle after the chromosome loss event due to rapid proteasome degradation of the α2 repressor (Laney *et al.* 2006) (Figure 1A).

The tester chromosome can in principle be any chromosome; however, loss of most native chromosomes in haploid or diploid strains is likely to result in lethality or reduced proliferation rate compared to the non-fluorescent euploid cells in the population, leading to an under-estimation of CIN. To solve this issue, we took advantage of the previously developed MC composed of the left arm of yeast Chromosome III (Chr III) and a very short right arm (Spencer *et al.* 1990). The MC carries 70 ORFs (Table S3) and the selectable nutrient marker (*LEU2*), thus minimizing significant fitness consequence of MC loss if cultured in nonselective media, as confirmed below (Figure 1D). We introduced the aforementioned *MATα* locus to the right arm of this highly telocentric MC (Figure 1A). The proximity of *MATα* locus to the centromere and its flanking by different DNA sequences from the bacteria plasmid pBR322 make it highly unlikely that the locus is lost through mitotic recombination (Spencer *et al.* 1990). In addition, by using the classical CTF assay the MC loss rate was estimated to be 7×10^−4^ per cell division (Warren *et al.* 2002) and whole MC loss was the predominant mechanism observed. As such, our MC-based qCTF assay is designed to report the frequency of whole chromosome loss and minimizes the complication from other genetic or fitness changes.

To validate various assumptions of the assay, we subjected the aforementioned qCTF strain grown in media nonselective for the MC to flow cytometry analysis. As expected, the vast majority of the population was negative for GFP fluorescence. A very small fraction (0.246 ± 0.008%, n = 8 experiments) exhibited a GFP fluorescence level ∼100-fold greater than the GFP^−^ population (Figure 1B). For negative and positive controls, this highly fluorescent population was not present from a parental strain where the 3×GFP tag was not inserted and was the dominant population in the parent strain containing the 3×GFP tag but without the added *MATα* locus (Figure S1A). We then sorted the GFP^+^ population using fluorescence-activated cell sorting and plated cells at single-colony density on plates with complete media (YPD) followed by replicating on leucine-deficient media. 97% of these colonies were Leu^-^, suggesting the loss of the MC (Figure S1B). Furthermore, 11 colonies were randomly picked and karyotyped by using the previously developed qPCR karyotyping assay (Pavelka *et al.* 2010). Unlike the MC-containing strain, which had two copies of Chr III left arm and 1 copy of the right arm, all 11 colonies contained only one copy of each of these, confirming MC loss (Figure 1C, Figure S1C). These analyses confirmed that GPF^+^ cells resulted mostly from MC loss.

### Quantitative performance of qCTF assay

To obtain quantitative measurement of CIN with qCTF assay in high throughput, we developed a mathematical formula for extrapolation of MC loss rate *m* from a simple experimental design. $m=1-2^{\beta -1}q$, where *q* is found from $\;q^{n}R_{n}=R_{0}+(2^{1-\beta }-q)(1-q^{n})/(1-q)$. This formula, a derivation of which is presented in the extended Materials and Methods, contains 4 independent parameters: $R_{0}=N_{0}^{-}/N_{0}^{+},$ ratio of number ($N_{0}^{-}$) of MC^−^ cells (GPF^+^ population) to number ($N_{0}^{+}$) of MC^+^ cells (GFP^-^ population) at t=0, $R_{n}=N_{n}^{-}/N_{n}^{+},$ ratio of number $N_{n}^{-}$ of MC^−^ cells to number $N_{n}^{+}$ of MC^+^ cells at time *t* after $n=k_{+}t/ln2$ divisions of MC^+^ cells with the growth rate $k_{+}$, and $\beta =T_{-}/T_{+},$ ratio of the doubling time $T_{-}$ for MC^−^ cells to the doubling time $T_{+}$ for MC^+^ cells. Using the sorted GPF^+^ and GFP^−^ populations, we compared the growth rates with or without MC present and found that MC loss had minimal impact on growth (Figure 1D). In this case β=1 and the formula simplifies to $m=1-{\left(\frac{1+R_{0}}{1+R_{n}}\right)}^{1/n}.$ Because in most cases, *m* << 1 the formula can be simplified even further to $m=\frac{R_{n}-R_{0}}{n(1+R_{n})}.$ For future adaptation of the qCTF assay to measurements of native chromosome loss rate, the linear plot can be used once relative growth rates under assay conditions are directly assessed with sorted GFP^+^ and GFP^−^ populations (Figure 1E).

**Figure 2:**
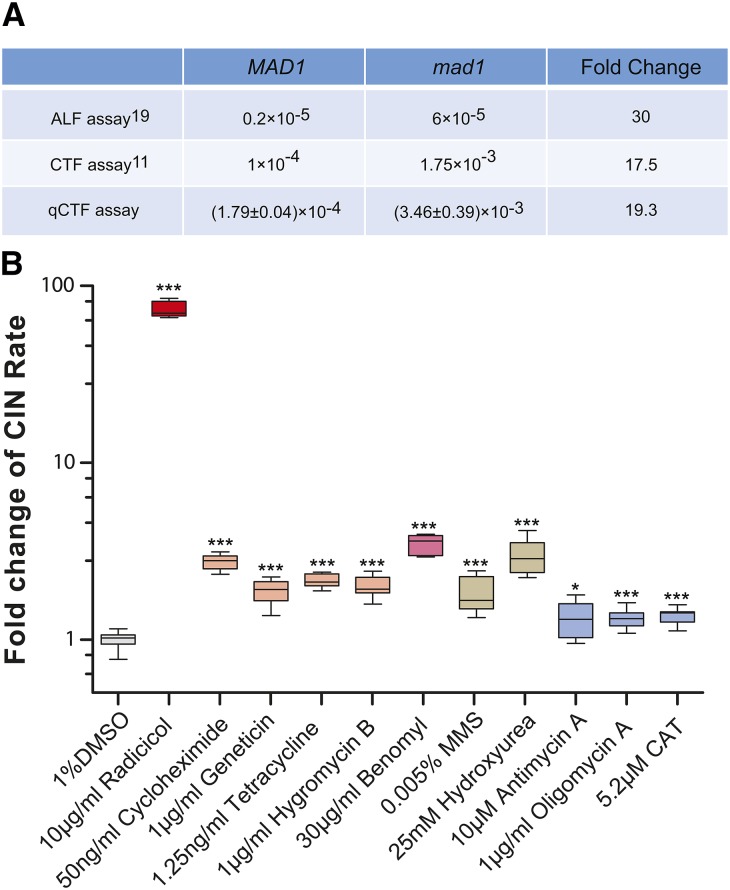
Quantitative performance of the quantitative chromosome transmission fidelity (qCTF) assay. (A) A table comparing the qCTF assay with the a-like faker (ALF) assay and CTF assay in detecting the rate of elevation of chromosome instability (CIN) caused by loss-of-function/deletion of *MAD1*. For qCTF assay, the data were shown as Mean ± SEM, n = 8. (B) Box-and-whisker plots showing the effect of different chemicals on CIN. *P*-values were calculated from the Mann–Whitney *U* test with n = 8. *P < 0.05; ***P < 0.001. DMSO, dimethyl sulfoxide; MMS, methyl methanesulfonate; CAT, carboxyatractyloside.

To test the performance of the qCTF assay, we applied it to a *MAD1* deletion strain. *MAD1* encodes a core component of the spindle assembly checkpoint (SAC), and we compared the obtained CIN rate with those reported previously for WT and *mad1Δ* by using ALF (Li and Murray 1991) and CTF assays (Spencer *et al.* 1990). For WT cells, the CIN rate measured with qCTF assay was within 2-fold of that estimated with CTF assay that scored for half sectors (Figure 2A) but ∼20 fold greater than that estimated from ALF. This was reasonable as the CTF assay also was based on loss of MC, whereas ALF assay was based on loss of the native Chr III. The different results between qCTF (or CTF) and ALF assay may be due to a difference in inherent transmission fidelity between MC and Chr III and/or the fact that MC-containing strain has an additional chromosome (MC) compared with the haploid strain used for ALF. Despite the difference in CIN rate obtained for corresponding strains between qCTF and ALF assays, the CIN rate change for *mad1Δ* mutant compared with WT as measured with these assays agreed well (Figure 2A). In this comparative experiment, one advantage of qCTF immediately became apparent: the qCTF assay examines millions of cells in minutes and is easily performed in replicates, enabling rigorous statistical analysis.

One potential industrial application of qCTF assay is for identification of aneugens, chemicals that trigger CIN and produce aneuploidy. qCTF could augment the widely-used Ames test for assessing carcinogenic potential (Ames *et al.* 1973). Radicicol, an Hsp90 inhibitor, was identified in a recent study as a potent aneugen for budding yeast by using the laborious colony-counting CTF assay (Chen *et al.* 2012). The qCTF assay confirmed radicicol’s dramatic ability to induce CIN (Figure 2B). Several translation inhibitors that also affect proteome homeostasis had milder but nonetheless significant enhancing effects on CIN (Figure 2B). Benomyl, a fungicide that destabilizes microtubules, was not found to elevate CIN at 30 µg/mL concentration with CTF assay (Chen *et al.* 2012); however, qCTF detected a 3.5-fold statistically significant increase in CIN (Figure 2B). We also tested the emerging idea that DNA replication stress causes CIN by examining the effect of hydroxyurea and methyl methanesulfonate by qCTF (Burrell *et al.* 2013). The result revealed a mild but significant increase in CIN (Figure 2B) at concentrations of these compounds that did not cause severe growth retardation. Finally, we found several inhibitors of mitochondrial function to cause a significant elevation of CIN (Figure 2B) at concentrations that did not arrest mitotic proliferation, supporting several previous studies implicating mitochondrial function in the fidelity of chromosome segregation (Dirick *et al.* 2014; Veatch *et al.* 2009).

### Quantitative profiling of genes that affect CIN when reduced in copy number

We envisioned two unique strengths with qCTF assay over existing assays for measuring CIN: enabling quantitative measurements of CIN with unprecedented sensitivity; and easy adaptability to high-throughput format. Taking advantage of both benefits we performed a genome-wide profiling of dosage-sensitive genes affecting CIN. Recent studies have shown that copy number alteration is a frequently occurring genetic change in cancer cells and that dosage imbalance of several highly conserved SAC proteins elevate CIN (Schvartzman *et al.* 2010; Zack *et al.* 2013). Identification of genes that affect CIN in a dosage-sensitive manner will facilitate the understanding of the molecular basis of aneuploidy-associated genome instability (Potapova *et al.* 2013). However, unlike strong loss of function mutations gene dosage effects are likely to be relatively subtle and requiring reliable and sensitive quantitative assays. Two previous studies (Choy *et al.* 2013; Strome *et al.* 2008) identified sets of genes that cause increased CIN when hemizygous. We refer to these genes as dcCIN genes. dcCIN genes from these two studies showed minimal overlap (Figure S2A) and most were not found to cause CIN when tested in haploid yeast with deletion or conditional alleles of these genes (Figure S2A). These discrepancies could be due to assay differences or the noise and semiquantitative nature of the previous assays, especially given that heterozygous deletion mostly confers a mild CIN phenotype. We therefore decided to screen for additional dcCIN genes by using qCTF assay in a high-throughput format.

**Figure 3:**
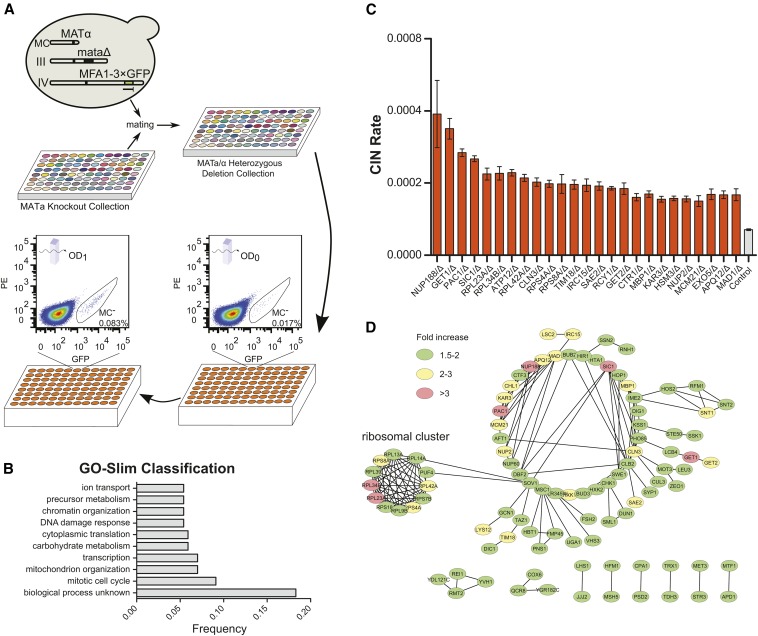
Genome-wide screen of nonessential yeast open-reading frame deletions for decreased in copy number (dcCIN) genes with the quantitative chromosome transmission fidelity (qCTF) assay. (A) A schematic representation of the strain generation and screen procedure for identifying dcCIN genes. In brief, qCTF strains hemizygous for each ORF deletion were obtained by mating as described in detail in the section *Materials and Methods*. OD and mini-chromosome negative (MC^−^) cell frequency (flow cytometry) were monitored at the beginning and end points for each culture grown in no-selective media for MC. Analysis details are given in the section *Materials and Methods*. (B) Gene Ontology Slim functional classification of dcCIN genes, showing only major groups with at least 10 genes with some highly similar groups being omitted. (C) A bar plot shows the rate of chromosome instability (CIN) of the top 25 dcCIN genes (orange bars). Data are shown as Mean ± SEM (n = 8). (D) Protein interaction network among dcCIN genes. Genes that cause different fold changes in CIN rate are differentially color coded as indicated.

The *MATa* locus of the WT qCTF yeast strain was deleted so that it would behave as an *MATα* haploid strain and can be mated to the nonessential *MATa* ORF deletion strain library, generating a library of diploid strains carrying heterozygous deletion of 4919 ORFs, of 4977 nonessential ORFs and containing the qCTF system in the genetic background (Figure 3A). We first used qCTF to validate 254 previously identified nonessential dcCIN genes. The assay was performed in 96-well sample format, where ∼0.3 million cells per replicate and eight replicates were analyzed for each strain at 0- and 24-hr time points after switching the cultures to nonselective media (see the section *Materials and Methods*). OD at each time point also was measured for each strain to estimate cell doubling number. We found 44% (111/254) of previously identified dcCIN genes were verified by qCTF to produce significant increase in CIN (fold change >1.5, *P* < 0.01) compared with a WT diploid control, and 87% of these caused elevation of CIN by less than twofold (Table S4). These results also validated the ability of qCTF to quantify small changes in CIN. We therefore went on to screen the remaining heterozygous deletion strains by qCTF. Because the primary screen of 4914 genes by flow cytometry was performed without replicates, strains with the top 105 highest and the top 101 lowest CIN rates were picked as candidate hits. These candidate hits were then rescreened with eight biological replicates, and 80 of them were validated to cause significant change in CIN (fold change >1.5, *P* < 0.01) compared with the WT control (Table S4).

The aforementioned analyses using qCTF produced a list of 186 high-confidence dcCIN genes. GO Slim Classification analysis revealed a subset of dcCIN genes belong to diverse processes such as DNA damage response, transcription, and cytoplasmic translation (Figure 3B). In addition, GO analysis revealed enrichment for genes regulating cell cycle, M phase, and the spindle pole body (Figure S3, A and B), as expected. Cell-cycle regulators among the top 25 hits included *SIC1* (a G1/S cyclin-dependent kinase inhibitor), *CLN3* (a G1 cyclin), and *MAD1* (a component of the SAC), *MBP1* (a transcription factor involved in G1/S regulation) (Figure 3C). Protein interaction network analysis (Franceschini *et al.* 2013) revealed that 95 of the dcCIN genes have interaction partners within the group, most notably the presence of a clear ribosome protein cluster (Figure 3D). This finding may be consistent with the effect of protein synthesis inhibitor on CIN (Figure 2B), suggesting a requirement for a balanced proteome in maintaining high chromosome segregation fidelity. Curiously, 162 dcCIN genes (Figure S2C) were not identified as CIN genes in haploid deletion screens (Stirling *et al.* 2011). For example, *NUP188* encodes a subunit of the inner ring of the nuclear pore complex and was not previously known to be involved in chromosome segregation; however, its human homolog was recently found to be required for chromosome alignment in mitosis in Hela cells (Itoh *et al.* 2013). Thus it may be useful to rescreen of the haploid ORF deletion library by qCTF assay. Interestingly, the nonessential dcCIN genes identified here were not found to be significantly enriched for core protein complexes (Figure S3E). This differs from the genes that are haploinsufficient for growth (Deutschbauer *et al.* 2005).

### Identification of genes that affect CIN when increased in copy number

**Figure 4:**
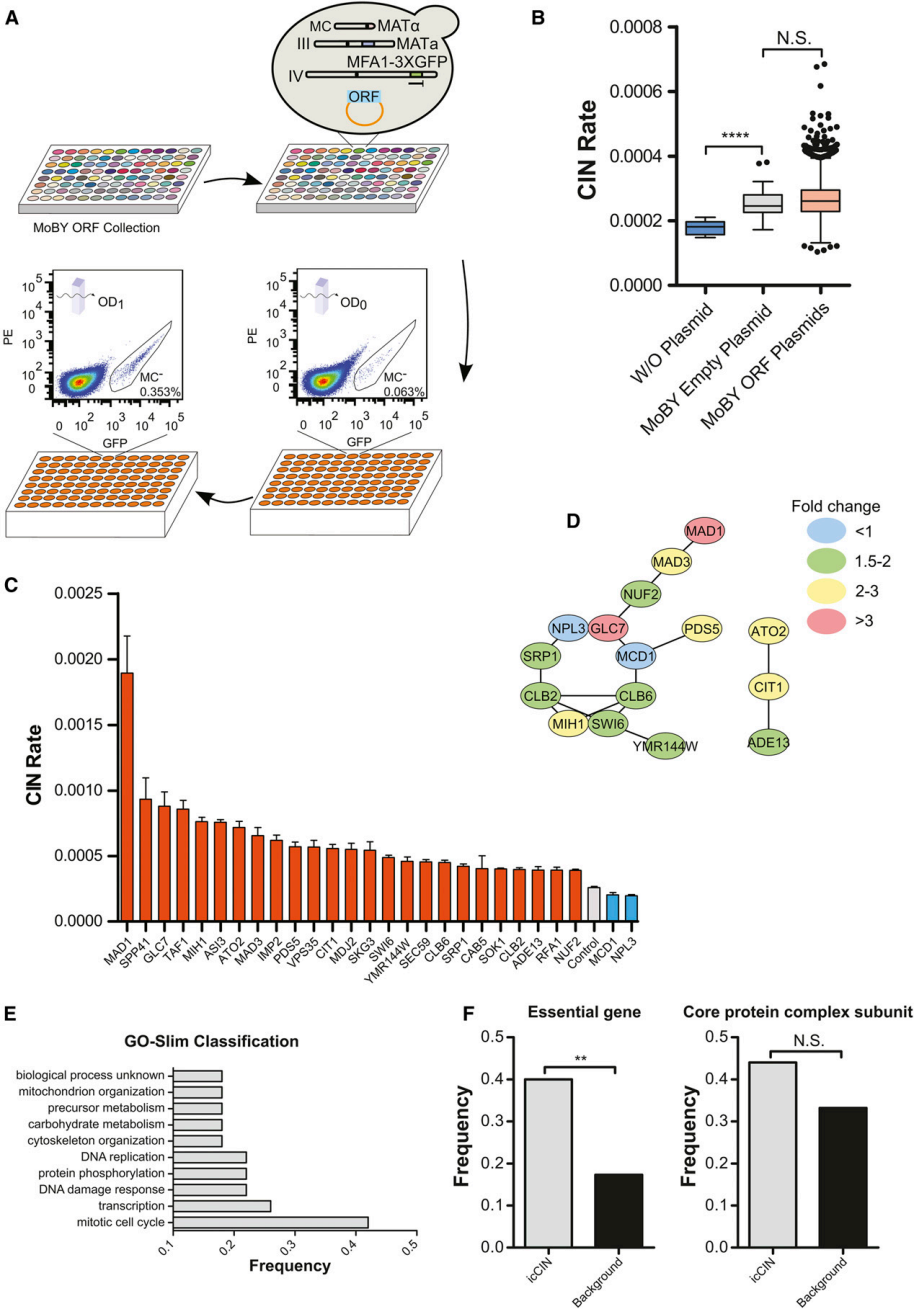
Genome-wide screen for increased in copy number (icCIN) genes with the quantitative chromosome transmission fidelity (qCTF) assay. (A) A schematic representation of the strain generation and screen procedure for a genome-wide screen for icCIN genes using the yeast MoBY open-reading frame (ORF) library. Experimental details are given in the section *Materials and Methods*. (B) Box-and-whisker plots comparing the CIN rate of the wild-type qCTF strain without (W/O) or with the MoBY empty control plasmid and qCTF strains with the MoBY ORF plasmids, n = 8 for WT qCTF strain; n = 58 for qCTF strain with control plasmid; n = 4932 for all MoBY ORFs in the primary screen. *P* value was calculated from the Mann-Whitney *U* test. ****P < 0.0001; N.S., nonsignificant. (C). Bar plots showing the rate of chromosome instability (CIN) of the 25 icCIN genes (orange bars) and two genes (blue bars) that suppressed basal CIN when increased in copy number. Data are shown as mean ± SEM (n= 8). (D) Protein interaction network among icCIN genes. Genes that cause different fold changes in CIN rate are differentially color coded as indicated. (E) Gene Ontology Slim functional classification of icCIN genes. Only groups with at least two genes are shown with some highly similar groups being omitted. (F) Bar plots comparing the frequency of essential gene and genes involved in core protein complex from icCIN genes or all MoBY plasmids screened (Background). *P* value was calculated from Fisher’s exact test. **P < 0.01; N.S., nonsignificant.

Gene or segmental chromosome amplification often lead to increased expression of the affected genes and is a frequently observed genetic change in evolving genomes (Santarius *et al.* 2010). It has been shown that increased expression of certain mitotic regulators disrupt chromosome transmission fidelity (Ricke *et al.* 2011; Ryan *et al.* 2012; Sotillo *et al.* 2007), but a comprehensive analysis of genes that elevates CIN at increased copy number has not be reported. To this end, we used qCTF assay in combination with the MoBY-ORF library containing 4956 yeast ORFs with their natural promoter and terminator (Ho *et al.* 2009) to perform a genome-wide profiling of genes for which a moderate increase in copy number affects CIN (referred as icCIN genes). The presence of a centromeric sequence on MoBY-ORF plasmids ensures that the copy number of the gene associated with each ORF is only increased by 1- to 3-fold. The individual centromeric MoBY-ORF plasmids were transformed in a high throughput manner into the qCTF yeast strain with an efficiency of 88%, resulting in 4392 strains (Figure 4A). The transformants were then subjected to high-throughput qCTF assay by flow cytometry as with the dcCIN screen, with the qCTF strain carrying an empty MoBY plasmid as the control. It is interesting to note that the CIN rate in this control strain is 1.4 fold higher (*P* < 0.0001) than the parental strain without the centromeric MoBY vector (Figure 4B), and additional centromeric plasmids leads to further increase in CIN (Figure S4). This finding suggests that extra centromeres in an otherwise-euploid genome elevate CIN.

The CIN rate distribution of all screened genes showed no significant difference when compared with the CIN rate distribution of the strain carrying the empty control plasmid, but outliers could be observed (Figure 4B). A total of 207 genes whose CIN rate ranked top 101 and bottom 106 were picked as candidate hits. To ensure the correct identity of the selected ORFs, the MoBY-ORF plasmids were recovered from these strains, amplified, and sequenced, revealing mislabeled or duplicated ORFs. To control for the mutagenic effect of plasmid transformation, 200 plasmids carrying confirmed genes were then retransformed into the qCTF strain and subjected to secondary validation using eight biological replicates, resulting in 25 high-confidence (fold change >1.5, *P* < 0.01) icCIN genes (Figure 4C). Protein interaction network analysis (Franceschini *et al.* 2013) revealed that more than 50% of icCIN genes interact with each other (Figure 4D). The major interaction network is composed mostly of genes involved in cell cycle regulation (Figure 4E), such as *CLB2* and *CLB6* (encoding two B-type cyclins), *MIH1* (encoding the budding yeast homolog of the Wee1 tyrosine kinase regulating Cdk1 activation), and SAC genes *MAD1* and *MAD*3. A smaller gene cluster included *ATO2*, *CIT1*, and *ADE13*, all encoding metabolic proteins. Enrichment for cell cycle genes was also indicated by GO analysis (Figure S3C). Proteins encoded by icCIN genes were enriched for nuclear and spindle components (Figure S3D). icCIN genes were also enriched for essential genes and showed higher though statistically insignificant increase in the fraction of core complex proteins (Figure 4F).

**Figure 5:**
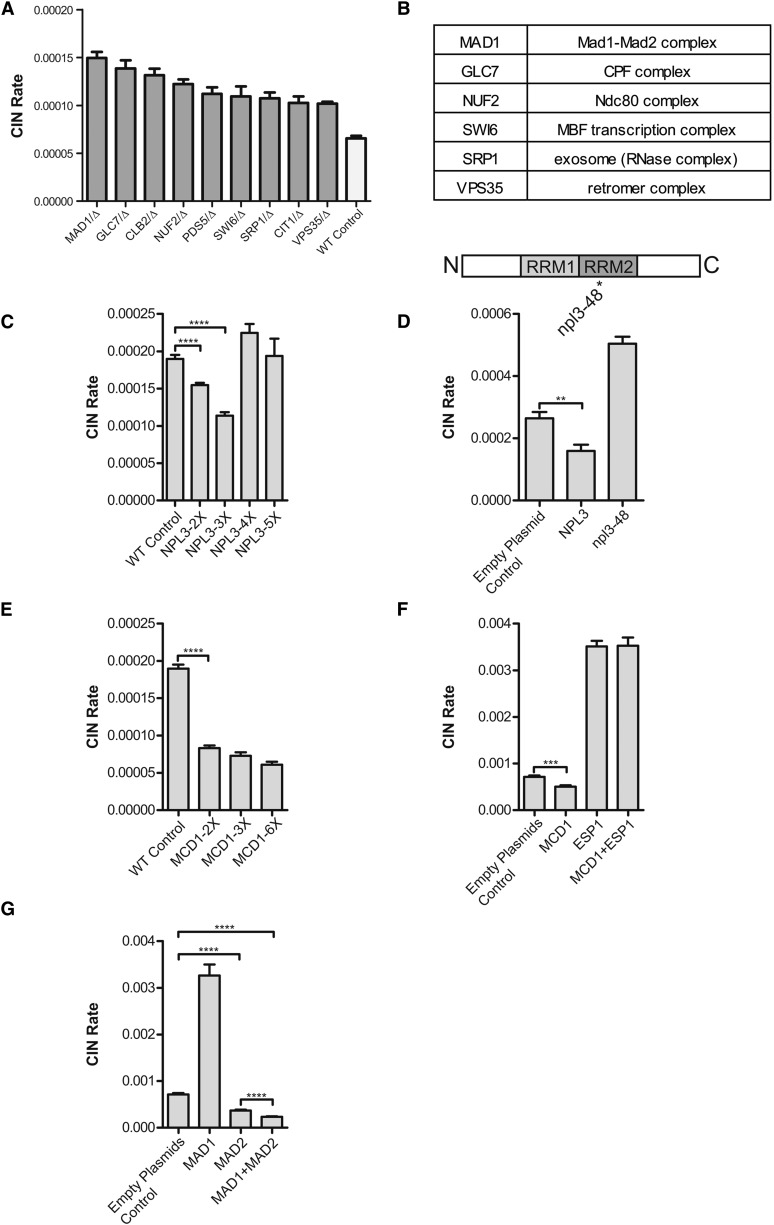
Characterization of chromosome instability (CIN) gene dosage sensitivity. (A) A bar plot shows CIN rate of nine strains each having heterozygous deletion of one icCIN gene (dark gray bar). These are thus two-way dosage-sensitive genes. Data are shown as mean ± SEM (n = 8). (B) A table displaying core complex involvement of six two-way dosage-sensitive CIN genes. (C) Bar plots showing the CIN rate of haploid strains with different copy number of genetically integrated *NPL3*. WT control has one native copy of *NPL3*. Data are shown as mean ± SEM, n = 8. *P* value was calculated from *t*-test. ****P < 0.0001. (D) Bar plots comparing the CIN rate of strains containing a blank centromeric plasmid, *NPL3* wild-type centromeric plasmid, and *NPL3* point mutant centromeric plasmid. Top panel indicates the position of the point mutation in the second RNA Recognition Motif (RRM2) of Npl3p. N (N terminus); C (C terminus). Mean ± SEM, n = 8. *P* value was calculated from *t*-test. **P < 0.01. (E) Bar plots showing the CIN rate of haploid strains with different copy number of genetically integrated *MCD1*. Data are shown as mean ± SEM, n = 8. *P* value was calculated from *t*-test. ****P < 0.0001. (F) Bar plots comparing CIN rate of strains containing two blank centromeric plasmids (Empty Plasmids Control), *MCD1* centromeric plasmid plus one blank centromeric plasmid (MCD1), *ESP1* centromeric plasmid plus one blank centromeric plasmid (ESP1), and *MCD1* centromeric plasmid plus *ESP1* centromeric plasmid (MCD1 + ESP1). Mean ± SEM, n = 8. *P* value was calculated from *t*-test. ***P < 0.001. (G) Bar plots comparing CIN rate of strains containing two blank centromeric plasmids (Empty Plasmids Control), *MAD1* centromeric plasmid plus one blank centromeric plasmid (MAD1), *MAD2* centromeric plasmid plus one blank centromeric plasmid (MAD2), and *MAD1* centromeric plasmid plus *MAD2* centromeric plasmid (MAD1 + MAD2). Mean ± SEM, n = 8. *P* value was calculated from *t*-test. ****P < 0.0001.

*MAD1* and *CLB2* represent overlapping hits from the dcCIN and icCIN screens (Figure S2D), suggesting the existence of two-way dosage sensitive CIN genes. To identify more of these genes, dcCIN analysis was performed for the 25 icCIN genes, which include 10 essential genes not previously included in the dcCIN screen but now deleted manually in a WT diploid qCTF strain. Altogether, 9 of 25 icCIN genes were found to be also dcCIN genes (Figure 5A). Interestingly, 6 of these genes encode components of core protein complexes (Figure 5B). For example, *GLC7*, encoding the catalytic subunit of protein phosphatase 1, is a stoichiometric subunit of the cleavage and polyadenylation factor complex, and Nuf2, is a component of the Ndc80 kinetochore complex. The fact that both increase and reduction in copy numbers of these genes enhanced CIN may support the idea that their dosage effect is due to an imbalanced protein stoichiometry rather than insufficiency or over-abundance in the activity of the encoded proteins (Papp *et al.* 2003).

### Two genes suppressing basal CIN when increased in copy number

A surprising finding of the icCIN screen, likely detected only as a result of the highly quantitative and sensitive nature of qCTF assay, was that two genes, *MCD1* and *NPL3*, each significantly reduced basal CIN rate (Figure 4C and Figure 5B) when present at increased copy numbers. *NPL3* encodes the yeast hnRNP that regulates mRNA transcription, processing and transport out of the nucleus (Lee *et al.* 1996). To more accurately evaluate the dosage effect of *NPL3* on CIN, we introduced different copies of this gene into the genome through homologous integration and analyzed the resulting strains with qCTF. This analysis showed a continuous decrease in CIN when *NPL3* copy number increased from 1 to 3 copies in the genome, but further increases began to reverse the CIN suppression phenotype, demonstrating quantitative dosage effect of *NPL3* on CIN (Figure 5C). To test whether CIN suppression requires the RNA-binding activity of Npl3p, we introduced a previously characterized point mutation (Lee *et al.* 1996) in the Npl3p RNA recognition motif into a centromeric plasmid and used qCTF to test its effect on CIN suppression. Interestingly, the *npl3-48* plasmid harboring point mutation S230P increased CIN in contrast to CIN suppression by WT *NPL3* plasmid (Figure 5D).

We also used homologous integration to introduce various copy numbers of *MCD1*, encoding the cohesion subunit cleaved by separase, into the yeast genome. In contrast to the results for *NPL3*, the decrease in CIN was roughly constant despite adding up to six copies of *MCD1* (Figure 5E), suggesting that *MCD1* dosage was no longer limiting after one additional copy. We considered the possibility that increased *MCD1* copy number could counter against some basal activity of Esp1 that disrupts proper chromosome cohesion. The addition of a centromeric plasmid containing *ESP1* caused a fourfold increase in CIN and overrode the effect of *MCD1* gain (Figure 5F). Finally based on a previous study that *MAD1* and *MAD2* gene dosage needs to be balanced for optimal checkpoint function (Barnhart *et al.* 2011), we introduced a MoBY plasmid carrying *MAD2* into the strain with the *MAD1* plasmid. Interestingly, whereas increased dosage of *MAD2* by itself moderately reduced CIN level, the dual presence of the *MAD1* and *MAD2* plasmid suppressed CIN to significantly below that of the basal level (Figure 5G). We note that none of the aforementioned gene copy number manipulations drastically altered the growth rate (Figure S5), and any change in cell cycle time was accounted for in our protocol of CIN rate measurement and calculation.

## Discussion

In this article, we have presented the development, validation and high-throughput application of a single cell−based and truly quantitative assay for assessment of chromosome transmission fidelity in budding yeast. A key design feature of qCTF is that a strong fluorescence signal from Mfa1p-3×GFP expression is gained in single cells upon removal of a transcriptional repressor carried on a particular chromosome that is lost during mitosis. Such events, although rare, can be captured and quantified in large cell populations by using flow cytometry. Combined with 96-well or possibly even 384-well sample plates and robotic plate loader, qCTF can be adapted for quantitative high-throughput analysis. We demonstrate the usefulness of qCTF by applying it to two genome-wide screens in yeast to identify genes that affect CIN in a dosage-sensitive manner. Our results show that qCTF is sensitive enough to detect the impact a small change in gene copy number can have on CIN, including not only the increase in CIN but also a decrease from the basal level. The assay is also sufficiently sensitive to detect increases in CIN caused by the presence of a centromeric plasmid, supporting the model of a recent study suggesting that CIN can be influenced by over-loading of the mitotic system with extra chromosome or centromeres to be segregated (Zhu *et al.* 2012).

The development of the qCTF assay provides a convenient and sensitive way to monitor CIN in budding yeast. However, it has several limitations. First, the current design does not rule out the possibility of mutations other than MC loss that contribute to the appearance of GFP^+^ cells. For example, point mutations in the α2 locus could contribute to the GFP^+^ population with MC being present. Thus, it is important to confirm MC loss in GFP^+^ cells of hits of interest in future studies. We therefore tested some our top hits and hits involved in DNA damage response (*NUP60*, *HSM3*, *CHK1*, *DUN1*, *RFA1*, *PDS5*), as the latter are more likely to be mutagenic, or chromatin organization (*HIR1*, *SNT1*, *HTA1*, *PUF4*) for the presence of MC after sorting for the GFP^+^ cells in selective media. We found that less than 2% of GFP^+^ cells from six icCIN hits still had a MC, except *SPP41* (15.5%) (Figure S6A). All but one (*HIR1*/*hir1Δ*) of the dcCIN hits tested had a level of GFP^+^ cells containing the MC at frequencies similar to or lower than that of the diploid control (Figure S6B). These results suggest that although the qCTF assay could be complicated by other genetic changes, MC loss is the predominant genetic change that leads to GFP expression in most strains examined. Second, the competition between MC^+^ and MC^−^ cells could bias the result of qCTF assay. In our high-throughput screen, we assumed similar growth rate between MC^+^ and MC^−^ cells to simplify the experimental design. To further validate this assumption, we compared the growth rates between MC^+^ and MC^−^ cells from several of our top hits. We found that for the five icCIN hits, MC^+^ cells grew slightly (5–10%) faster than MC^-^ cells, suggesting a slight underestimation of CIN rate (Figure S6C). For the three tested dcCIN hits, the differences in growth rates of these pairs were insignificant (Figure S6D). Third, the qCTF assay only reports the loss of the short and telocentric MC, but not a natural yeast chromosome. The stability of natural chromosomes could be affected by *cis*-acting elements such as centromeric sequence (Kumaran *et al.* 2013). Indeed, in a previous dcCIN gene screen using a different yeast MC, it was found that 85% of the hits resulted in greater than twofold increase in chromosome V loss rate (Strome *et al.* 2008).

Despite the aforementioned caveats, qCTF can still be a quantitative tool for probing molecular pathways controlling mitosis and chromosome segregation. It also can be useful in industrial settings for quantitative evaluating the aneugneic potential of environmental chemicals or pharmaceutical products. Likewise, the influence of natural products with anticancer therapeutic potential on CIN also can be evaluated using qCTF. Although our current qCTF assay is limited to tracking the segregation of an MC in yeast, we envision further expansion of the concept in two directions. First, the assay can be adapted to measurement of loss frequencies for any native chromosomes in a diploid or polyploidy yeast genome by inserting the repressor gene at a centromere-proximal location of the chromosome of interest, and then obtaining the growth rates of the yeast population before and after the loss of a copy of this chromosome (the latter can be sorted with fluorescence-activated cell sorting) to allow chromosome loss rate calculation. Such an adaptation of the assay may provide new insights into the relation between chromosome properties and transmission fidelity. Second, the assay may be adaptable to metazoan cells and organisms using appropriate repressor-reporter systems following similar design principles of the yeast qCTF assay. Such an assay has the potential to be superior in sensitivity, quantitation, and applicability to high throughput analysis compared with existing methods such as fluorescence in-situ hybridization or spectral karyotypin−based detection of chromosome numerical abnormality.

It is reassuring that the hits obtained from both screens performed with qCTF are enriched for cellular processes known to directly affect CIN, such as cell-cycle regulation and mitosis (Figure S3, A−D). However, our dcCIN hits overlap only slightly with previously CIN screens that used ORF deletion or temperature-sensitive mutant strains (Figure S2C). This may in part be explained by the fact that the previous screens were not aimed at finding dosage-sensitive genes but rather genes whose complete absence or inactivation would elevate CIN. Furthermore, inactivation of an essential gene could lead to cell-cycle arrest or rapid cell death, which would prevent growth or colony-based detection of chromosome loss events. It would thus be worthwhile in the future to use qCTF assay to re-profile yeast gene deletion or conditional loss-of-function libraries. We also noted that qCTF validated fewer than half of the hits from previous screens for genes that elevate CIN upon dosage reduction, whereas 40% (74/186) of the dcCIN hits, including many known to function in mitosis and cell cycle regulation, were missed in previous screens. This is likely to be explained by subtle effects of small gene dosage variation compounded by the difficulty in quantification associated with previous assays.

Our icCIN screen is the first of its kind to search for genes affecting CIN when increased in copy number and is distinct from previous work that uses the Gal-promoter (Ouspenski *et al.* 1999), which causes gross and deregulated overexpressions of ORFs. The centromere present on the MoBY plasmid helps to maintain the transgene at a low copy number and the presence of the native promoter in front of each ORF maintain the normal transcriptional regulation of the respective gene, which is often important for cell cycle regulators. The top hit from this screen is *MAD1*, encoding a highly conserved SAC kinase that forms stoichiometric complex with another conserved SAC protein Mad2. It was shown previously that a balanced Mad1/Mad2 dosage is critical for check point function and proper completion of mitosis. This requirement may also account for elevated CIN observed in aneuploid strains with higher Chr VII to Chr X ratios (Barnhart *et al.* 2011; Zhu *et al.* 2012). It is intriguing that the next three greatest-impact icCIN genes, *SPP41*, *GLC7* and *TAF1*, all encode proteins with known roles in transcription and RNA processing. Further, one of the two genes that suppress CIN when increased in dosage, *NPL3*, is a major RNA binding protein important for pre-mRNA splicing and transport. *GLC7*, *TAF1*, and *NPL3* gene deletion also were shown previously to elevate CIN (Francisco *et al.* 1994; Stirling *et al.* 2011; Wahba *et al.* 2011). One potential role hypothesized for Npl3 is to limit the concentration of RNA in the nucleus, thereby preventing the formation of DNA:RNA hybrids (R-loops), which can lead to DNA damage and CIN (Aguilera and García-Muse 2012). A recent study demonstrated a direct involvement of Npl3-containing heterogeneous ribonucleoprotein particle complex that suppresses CIN in preventing R-loop stabilization (Santos-Pereira *et al.* 2013), consistent with our result demonstrating that introducing a point mutation in the RNA recognition motif of Npl3 not only reversed the CIN-suppressing effect but led to elevated CIN. In addition to this potential mechanism, these RNA regulatory genes could also exert their effects on CIN by influencing the timing or the level of expression of cell cycle regulators such as G1 or mitotic cyclins.

Our analyses using the qCTF assay revealed three conditions that could suppress basal CIN level through different mechanisms based on the known functions of these genes - increased copy numbers of *NPL3*, *MCD1*, or both *MAD1* and *MAD2* together. This raises the question as to why the basal CIN level for WT euploid cells growing under stress-free condition is not minimized, since the reduction of basal CIN rate can be so easily accomplished through small increase in the expression of different genes. A potential answer to this question is that the basal error rate for mitosis might have been optimized rather than minimized to ensure a high-level genome stability and also permit adaptability by maintaining a minute level of karyotypic variants in the population. Emerging evidence from different organisms have shown that aneuploid karyotypes usually confer decreased fitness under stress-free condition, but in the presence of stress, the significant phenotypic variation conferred by karyotype diversity provides the substrate for evolutionary selection of adaptive variants (Ni *et al.* 2013; Pavelka *et al.* 2010; Selmecki *et al.* 2006). This dichotomy implies the possibility of an optimal error rate small enough to be well tolerated without stress but large enough to endow the population a sufficient level of inherent adaptability to cope with exposure to acute stress.

## Supplementary Material

Supporting Information
