# Supplementary material for: Single-Cell Based Quantitative Assay of Chromosome Transmission Fidelity
Source: G3 (Bethesda). 2015 Mar 30;5(6):1043–56. doi: 10.1534/g3.115.017913 (PMC4478535; doi:10.1534/g3.115.017913)
Supplement: Supporting Information [file supp_5_6_1043__index.html]

Single-Cell Based Quantitative Assay of Chromosome Transmission Fidelity — Supporting Information 

# Single-Cell Based Quantitative Assay of Chromosome Transmission Fidelity

## Supporting Information for Zhu *et al.*, 2015

**Files in this Data Supplement:**

- Supporting Information - Figures S1-S6, File S1, and Tables S1-S4 (PDF, 1 MB)
- Figure S1 - Additional validation assays for qCTF. (PDF, 1 MB)
- Figure S2 - Comparison of CIN gene hits from screens in this and published studies. (PDF, 145 KB)
- Figure S3 - GO analysis of dosage-sensitive genes identified in qCTF screens. (PDF, 145 KB)
- Figure S4 - Extra centromeres in qCTF strain elevate CIN. (PDF, 127 KB)
- Figure S5 - Effects of gene dosage on cell doubling time. (PDF, 128 KB)
- Figure S6 - Additional validation results for the qCTF assay. (PDF, 136 KB)
- File S1 - Extended Materials and Methods (PDF, 897 KB)
- Table S1 - (a) Genotype of yeast strains. (b) Plasmids used in the study. (.xlsx, 11 KB)
- Table S2 - Primers used in this study. (.xlsx, 9 KB)
- Table S3 - ORFs on the mini-chromosome (MC). (.xlsx, 13 KB)
- Table S4 - (a) dcCIN genes identified with Chromosome V loss assay in Strome *et al.*, 2008. (b) dcCIN genes identified with ALF assay in Choy *et al.*, 2013. (c) dcCIN identified in current study. (d) all dcCIN genes. (.xlsx, 40 KB)
