# Supplementary material for: Single-Cell Based Quantitative Assay of Chromosome Transmission Fidelity
Source: G3 (Bethesda). 2015 Mar 30;5(6):1043–56. doi: 10.1534/g3.115.017913 (PMC4478535; doi:10.1534/g3.115.017913)
Supplement: Supporting Information [file supp_g3.115.017913_017913SI.pdf]

G3 · Genes | Genomes | Genetics

Single-Cell Based Quantitative Assay of Chromosome Transmission Fidelity

Jin Zhu[1], Dominic Heinecke[1], Wahid Mulla[1, 2], William D. Bradford[1], Boris Rubinstein[1], Andrew Box[1], Jeffrey S. Haug[1] and Rong Li[1, 2, 3]

[1]Stowers Institute for Medical Research, 1000 East 50th Street, Kansas City, MO 64110, USA

[2]Department of Molecular and Integrative Physiology, University of Kansas Medical Center, 3901 Rainbow Boulevard, Kansas City, KS 66160, USA

[3]Correspondence: rli@stowers.org

DOI: 10.1534/g3.115.017913

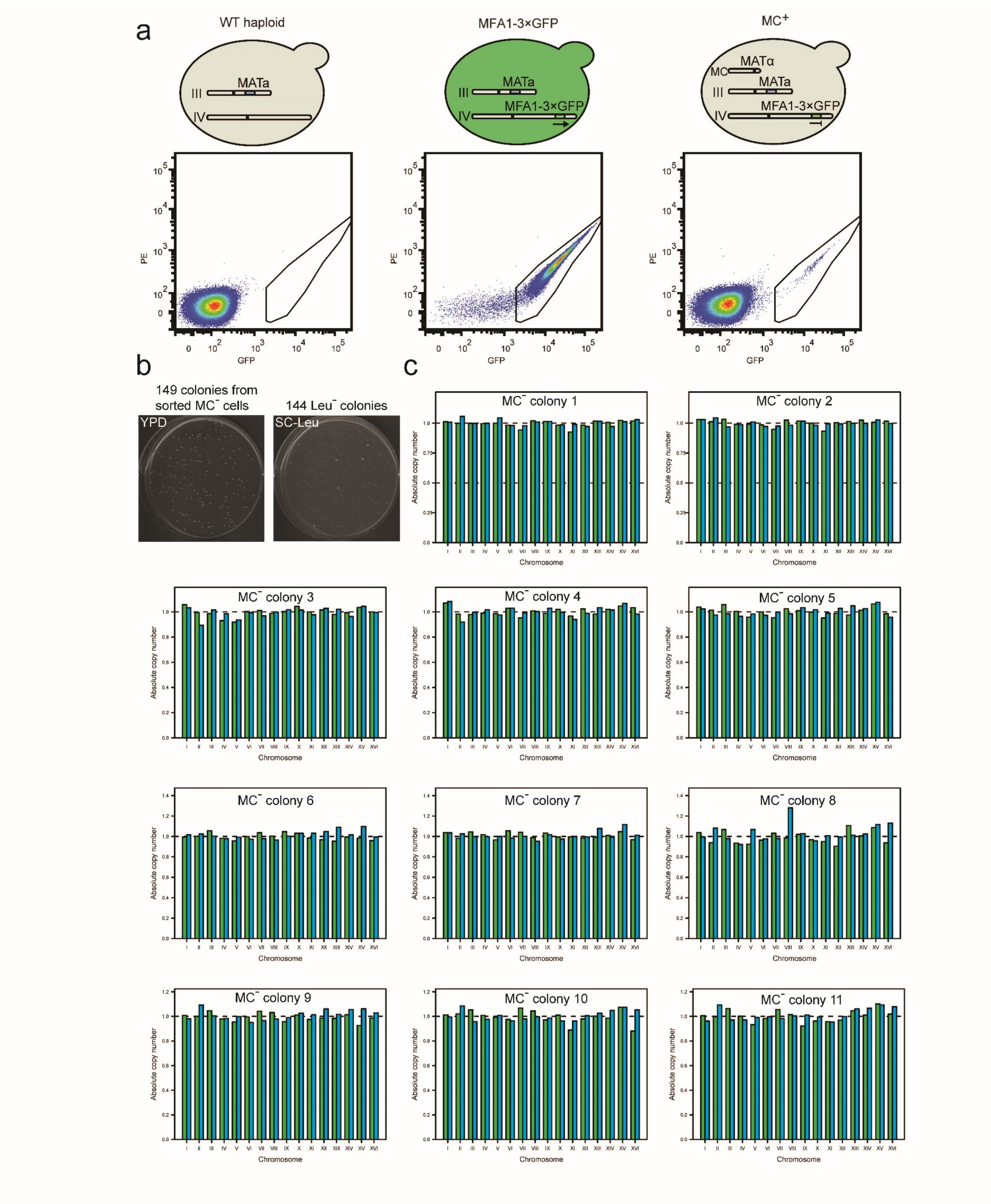

Figure S1 Additional validation assays for qCTF.

a. Flow cytometry analysis of qCTF parental strain (left), a negative control strain without GFP tagging (right), or positive control strain with MFA1-3×GFP tagging but no MC that contains the $\alpha 2$ repressor (middle).

b. Scanned image of an YPD (yeast extract peptone dextrose) plate containing colonies from FACS sorted MC^- cells. Another image shows the colony growth of the YPD plate replica-plated on to a SC-Leu plate selecting for MC, showing 97% (144/149) had lost the *LEU2* gene also carried on MC.

c. Karyotype of 11 randomly selected colonies from the YPD plate in b.

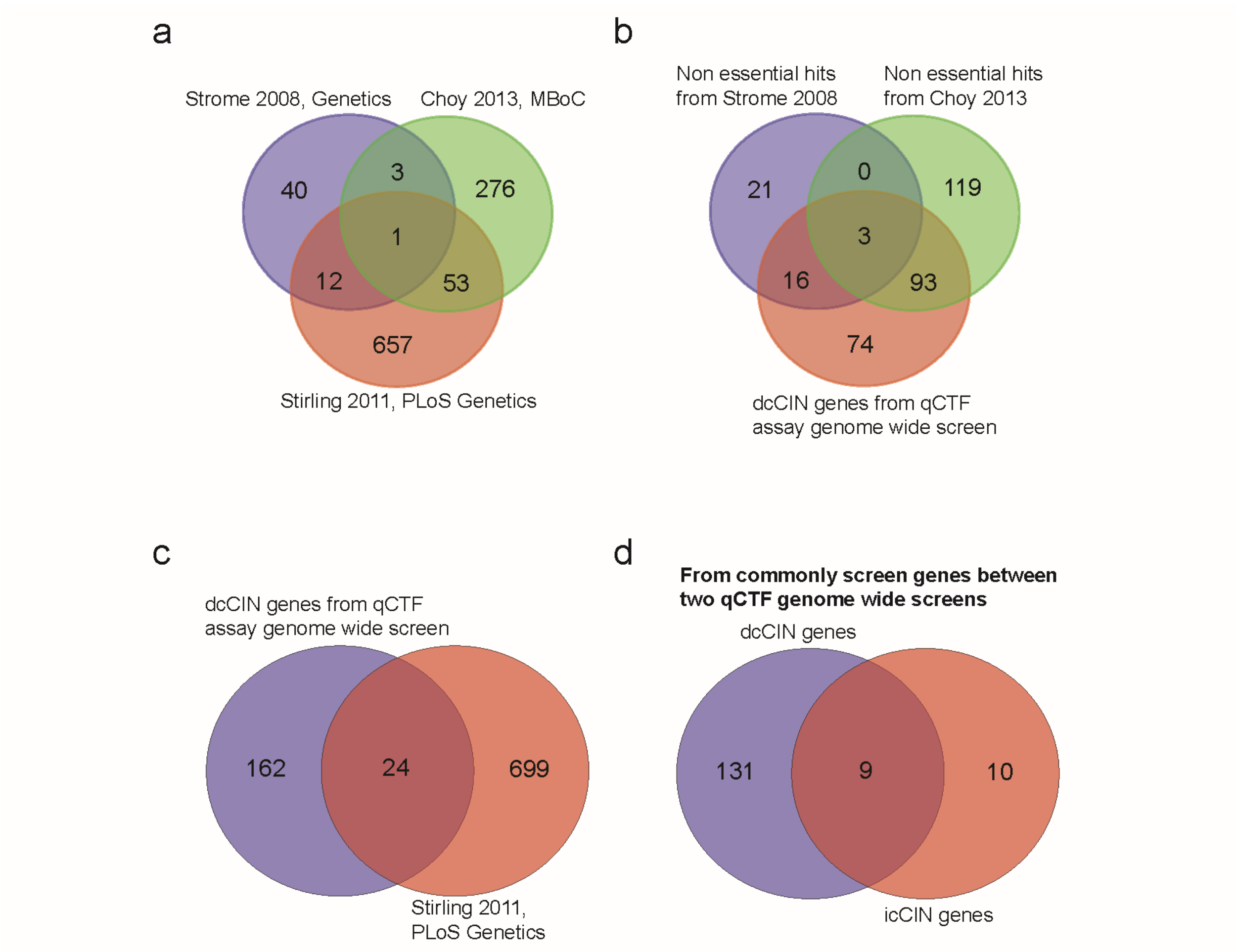

Figure S2 Comparison of CIN gene hits from screens in this and published studies

a-d. Published dcCIN datasets were from Strome et al. 2008[27] and Choy et al. 2013[28]. And published deletion or conditional CIN genes were from Stirling et al. 2011[10]. Because different screens included different sets of yeast genes, only hits from the common-screened genes are subjected to this analysis.

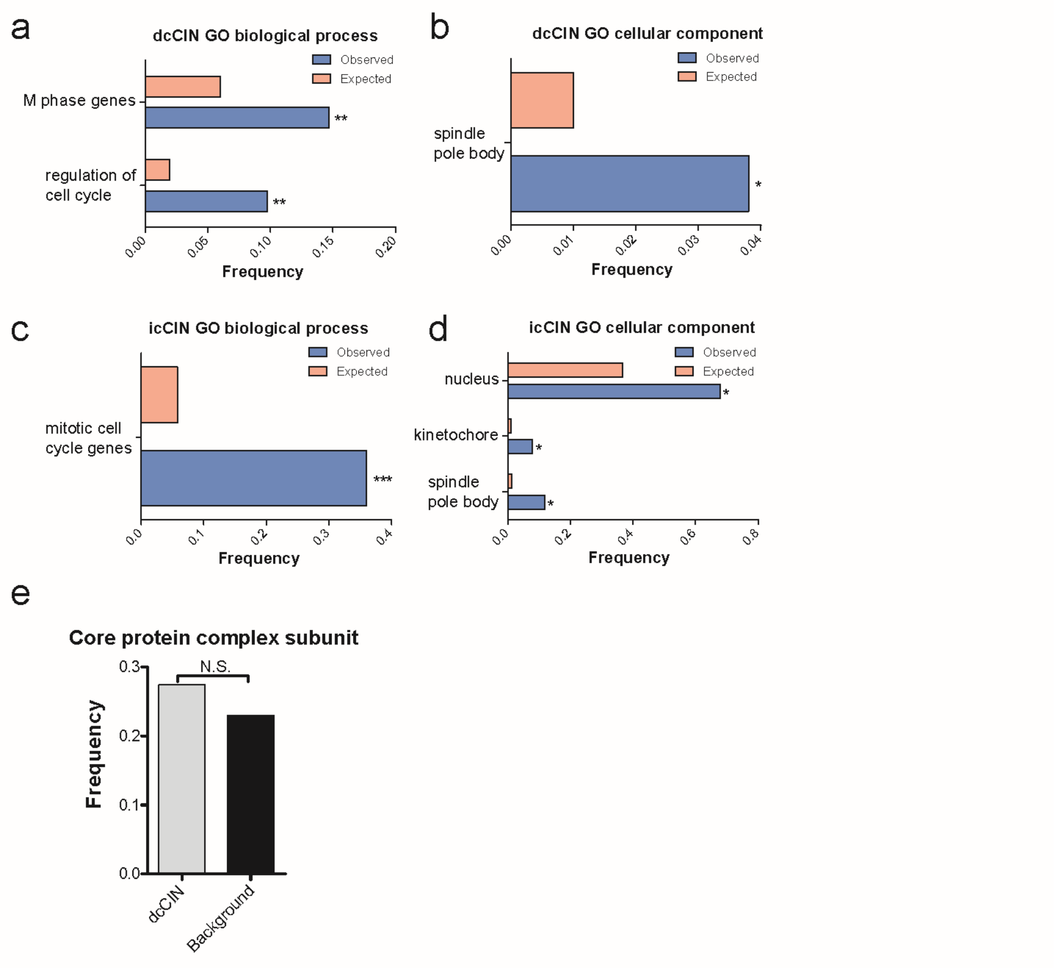

Figure S3 GO analysis of dosage-sensitive genes identified in qCTF screens

a-d. Gene Ontology enrichment analysis for 193 dcCIN genes and 25 icCIN genes. P value was calculated from Hypergeometric test with multiple test adjustment. One asterisk, $p < 0.05$; two asterisks, $p < 0.01$; three asterisks, $p < 0.001$.

e. A bar plot shows the frequency of genes involved in core protein complexes from dcCIN genes or non-essential genes screened (Background). P value of 0.184 was calculated from Fisher's exact test.

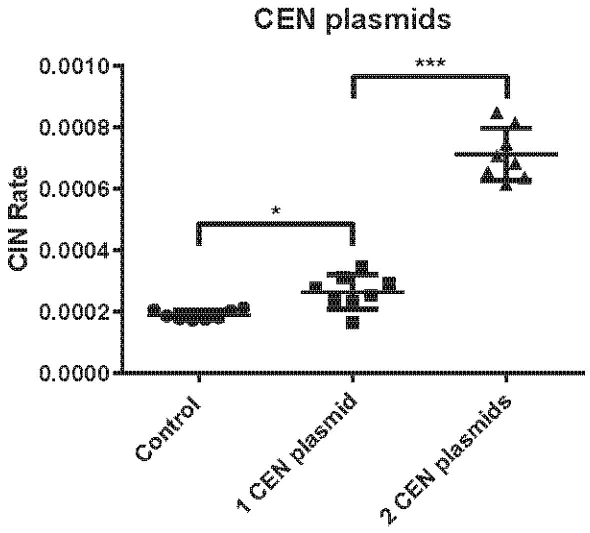

Figure S4 Extra centromeres in qCTF strain elevate CIN

A box plot shows the CIN rate change of the haploid qCTF strain after transforming with one centromeric plasmid (CEN-URA) or two centromeric plasmids (CEN-URA, CEN-TRP). P value was calculate from Mann Whitney test with n=8. One asterisk, $p < 0.05$; three asterisks, $p < 0.001$.

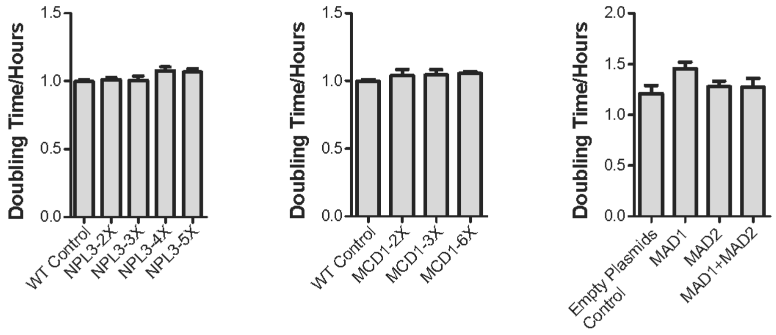

Figure S5   Effects of gene dosage on cell doubling time

Bar plots showing the doubling time of strains used in Fig. 5. Data are shown as Mean ± SEM, n=6.

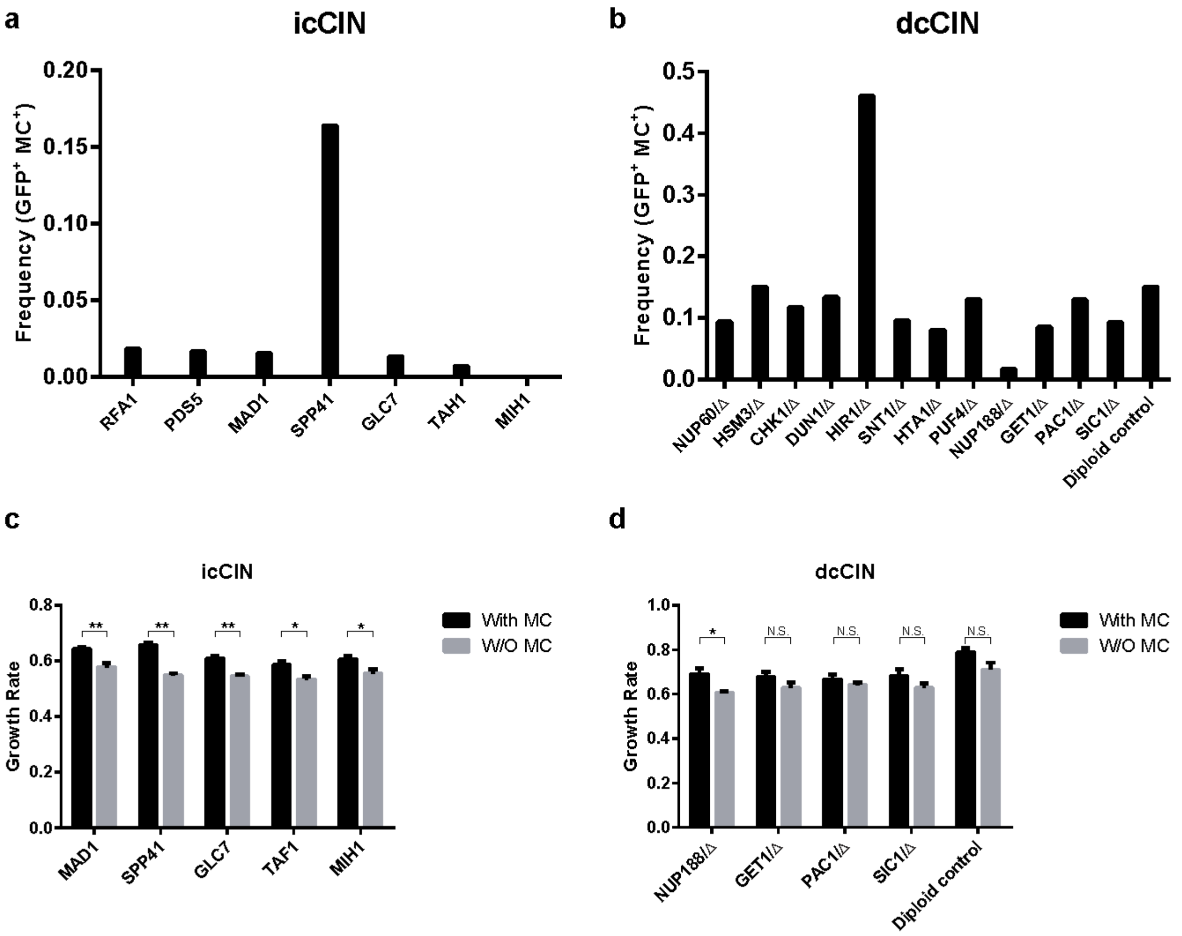

Figure S6 Additional validation results for the qCTF assay

a-b. Bar plots showing genetic changes other than MC loss that contribute to GFP⁺ cell populations in selected hits from icCIN and dcCIN gene screens. Experiment was performed as described in Figure S1 b.

c-d. Bar plots comparing growth rates of MC⁺ versus MC⁻ in some of the hit strains. Experiment was performed as described in Figure 1d. Data were shown as Mean ± SEM, n=8. P value was calculate from Mann Whitney. One asterisk, $p < 0.05$; two asterisks, $p < 0.01$; N.S.: non-significant.

Tables S1-S4

Available for download as Excel files at www.g3journal.org/lookup/suppl/doi:10.1534/g3.115.017913/-/DC1

Table S1 (a) Genotype of yeast strains. (b) Plasmids used in the study

Table S2 Primers used in this study

Table S3 ORFs on the mini-chromosome (MC)

Table S4 (a) dcCIN genes identified with Chromosome V loss assay in Strome *et al.*, 2008. (b) dcCIN genes identified with ALF assay in Choy *et al*., 2013. (c) dcCIN identified in current study. (d) all dcCIN genes.

File S1: Extended Materials and Methods

Available for download as a PDF file at www.g3journal.org/lookup/suppl/doi:10.1534/g3.115.017913/-/DC1
