# Supplementary material for: Single-Cell Based Quantitative Assay of Chromosome Transmission Fidelity
Source: G3 (Bethesda). 2015 Mar 30;5(6):1043–56. doi: 10.1534/g3.115.017913 (PMC4478535; doi:10.1534/g3.115.017913)
Supplement: Supporting Information [file supp_g3.115.017913_FigureS1.pdf]

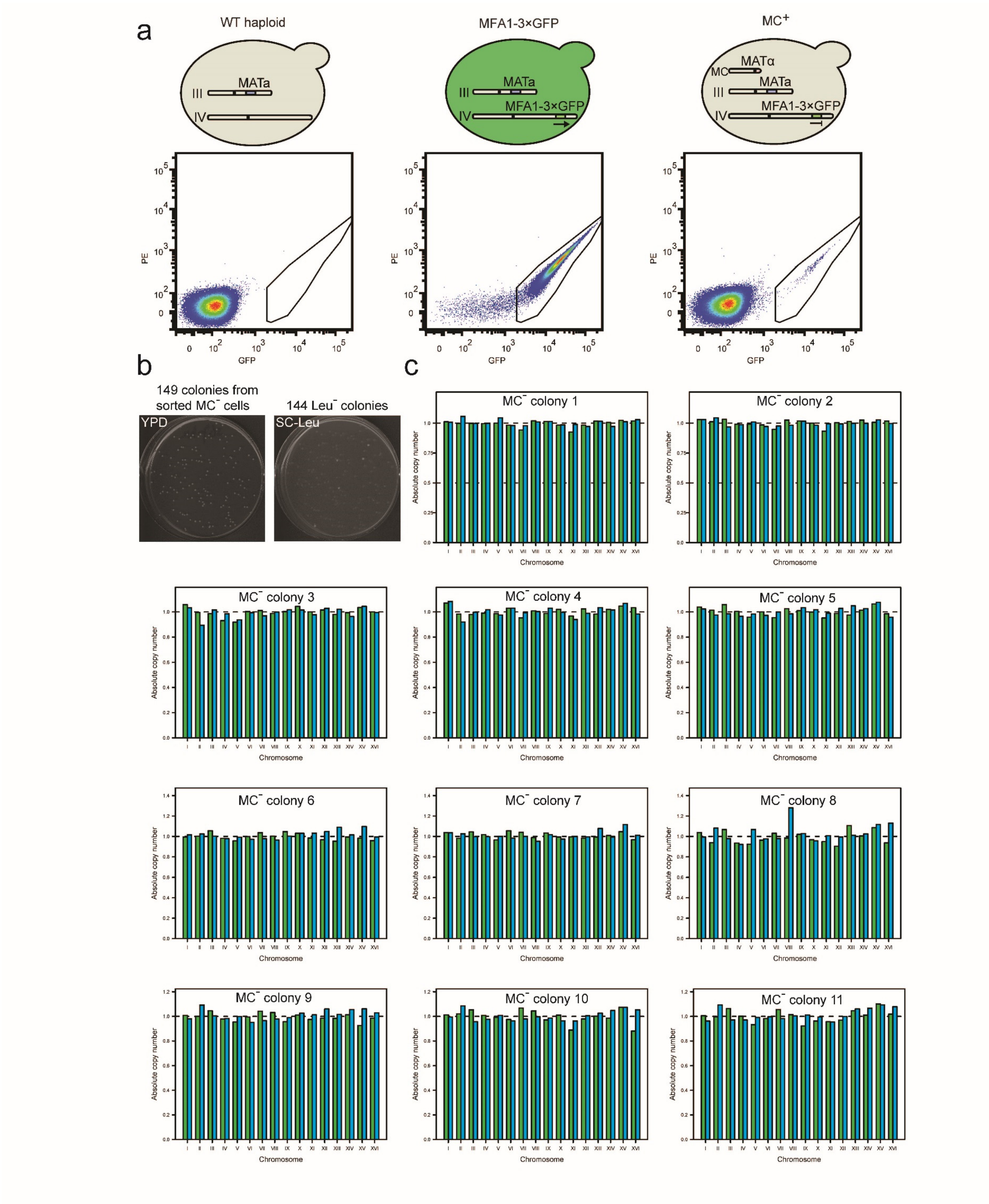

Figure S1 Additional validation assays for qCTF.

a. Flow cytometry analysis of qCTF parental strain (left), a negative control strain without GFP tagging (right), or positive control strain with MFA1-3×GFP tagging but no MC that contains the α2 repressor (middle).

b. Scanned image of an YPD (yeast extract peptone dextrose) plate containing colonies from FACS sorted MC^- cells. Another image shows the colony growth of the YPD plate replica-plated on to a SC-Leu plate selecting for MC, showing 97% (144/149) had lost the *LEU2* gene also carried on MC.

c. Karyotype of 11 randomly selected colonies from the YPD plate in b.
