# Supplementary material for: Single-Cell Based Quantitative Assay of Chromosome Transmission Fidelity
Source: G3 (Bethesda). 2015 Mar 30;5(6):1043–56. doi: 10.1534/g3.115.017913 (PMC4478535; doi:10.1534/g3.115.017913)
Supplement: Supporting Information [file supp_g3.115.017913_FigureS2.pdf]

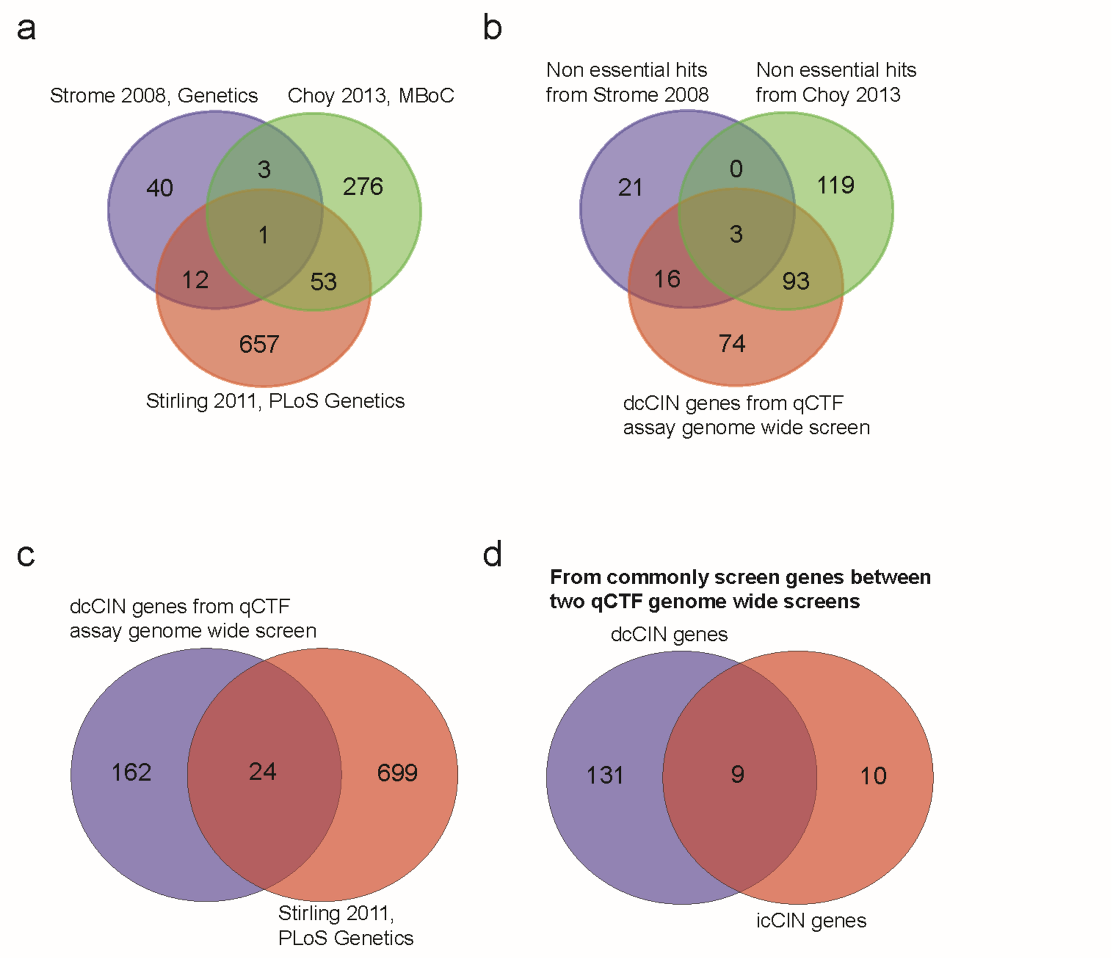

Figure S2 Comparison of CIN gene hits from screens in this and published studies

a-d. Published dcCIN datasets were from Strome et al. 2008[27] and Choy et al. 2013[28]. And published deletion or conditional CIN genes were from Stirling et al. 2011[10]. Because different screens included different sets of yeast genes, only hits from the common-screened genes are subjected to this analysis.
