# Supplementary material for: Single-Cell Based Quantitative Assay of Chromosome Transmission Fidelity
Source: G3 (Bethesda). 2015 Mar 30;5(6):1043–56. doi: 10.1534/g3.115.017913 (PMC4478535; doi:10.1534/g3.115.017913)
Supplement: Supporting Information [file supp_g3.115.017913_FigureS3.pdf]

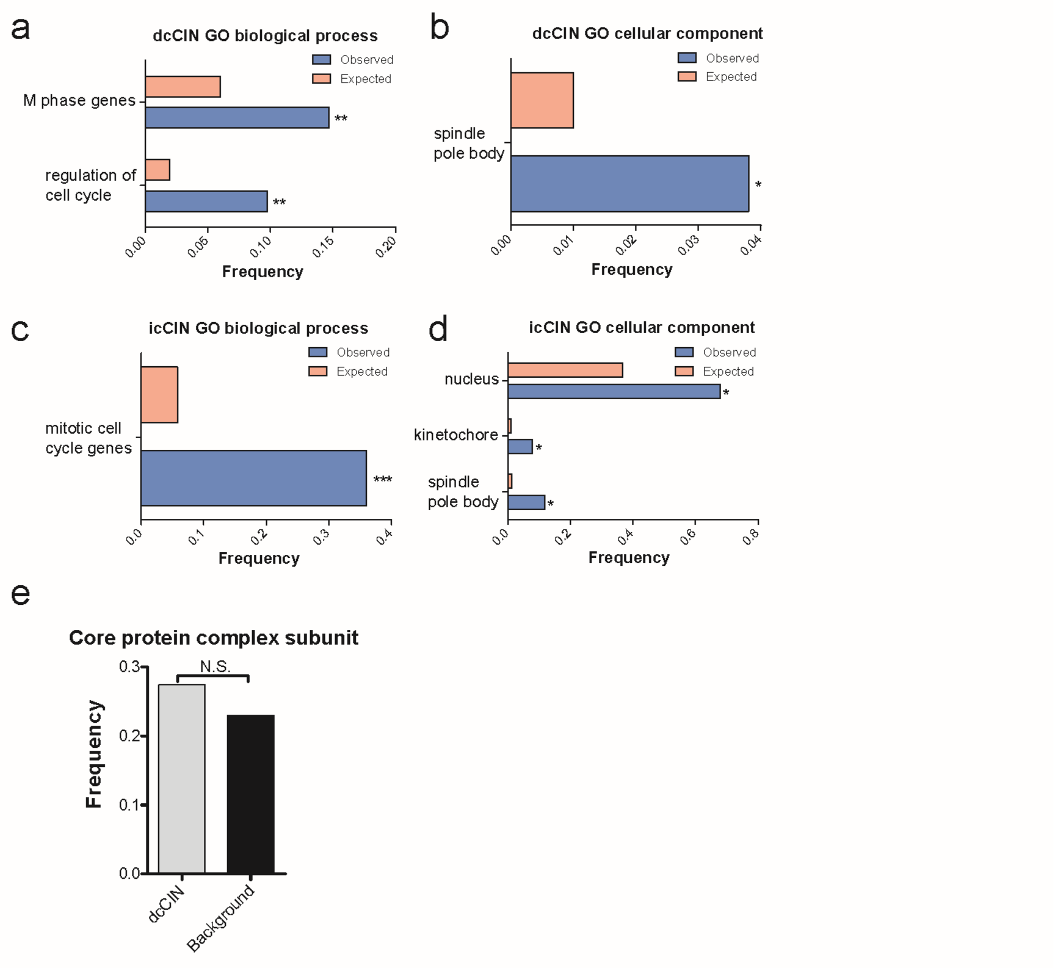

Figure S3   GO analysis of dosage-sensitive genes identified in qCTF screens

a-d. Gene Ontology enrichment analysis for 193 dcCIN genes and 25 icCIN genes. P value was calculated from Hypergeometric test with multiple test adjustment. One asterisk, $p < 0.05$; two asterisks, $p < 0.01$; three asterisks, $p < 0.001$.

e. A bar plot shows the frequency of genes involved in core protein complexes from dcCIN genes or non-essential genes screened (Background). P value of 0.184 was calculated from Fisher's exact test.
