# Supplementary material for: Single-Cell Based Quantitative Assay of Chromosome Transmission Fidelity
Source: G3 (Bethesda). 2015 Mar 30;5(6):1043–56. doi: 10.1534/g3.115.017913 (PMC4478535; doi:10.1534/g3.115.017913)
Supplement: Supporting Information [file supp_g3.115.017913_FigureS4.pdf]

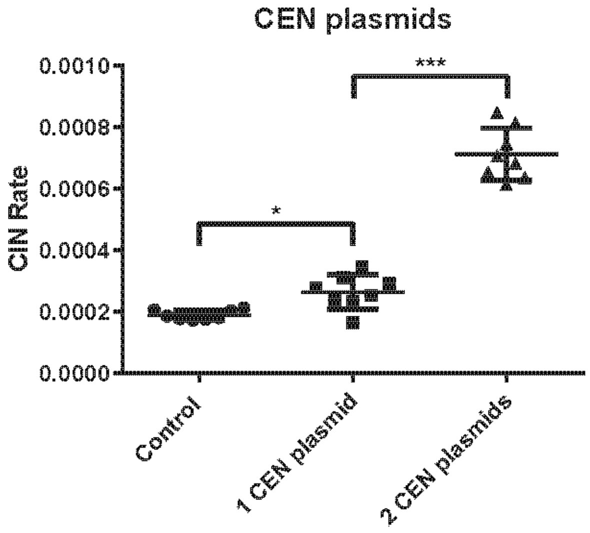

Figure S4 Extra centromeres in qCTF strain elevate CIN

A box plot shows the CIN rate change of the haploid qCTF strain after transforming with one centromeric plasmid (CEN-URA) or two centromeric plasmids (CEN-URA, CEN-TRP). P value was calculate from Mann Whitney test with n=8. One asterisk, $p < 0.05$; three asterisks, $p < 0.001$.
