# Supplementary material for: Single-Cell Based Quantitative Assay of Chromosome Transmission Fidelity
Source: G3 (Bethesda). 2015 Mar 30;5(6):1043–56. doi: 10.1534/g3.115.017913 (PMC4478535; doi:10.1534/g3.115.017913)
Supplement: Supporting Information [file supp_g3.115.017913_FigureS5.pdf]

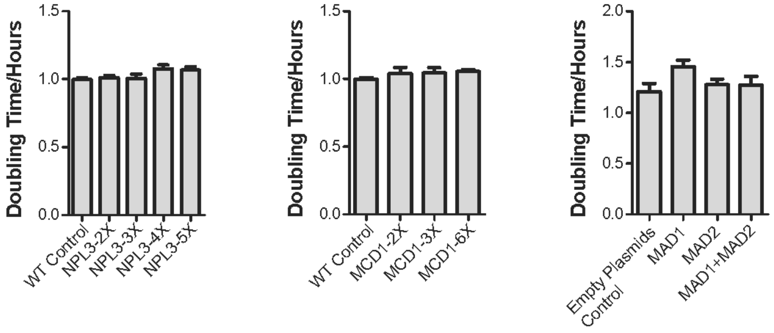

Figure S5 Effects of gene dosage on cell doubling time

Bar plots showing the doubling time of strains used in Fig. 5. Data are shown as Mean ± SEM, n=6.
