# Supplementary material for: Single-Cell Based Quantitative Assay of Chromosome Transmission Fidelity
Source: G3 (Bethesda). 2015 Mar 30;5(6):1043–56. doi: 10.1534/g3.115.017913 (PMC4478535; doi:10.1534/g3.115.017913)
Supplement: Supporting Information [file supp_g3.115.017913_FigureS6.pdf]

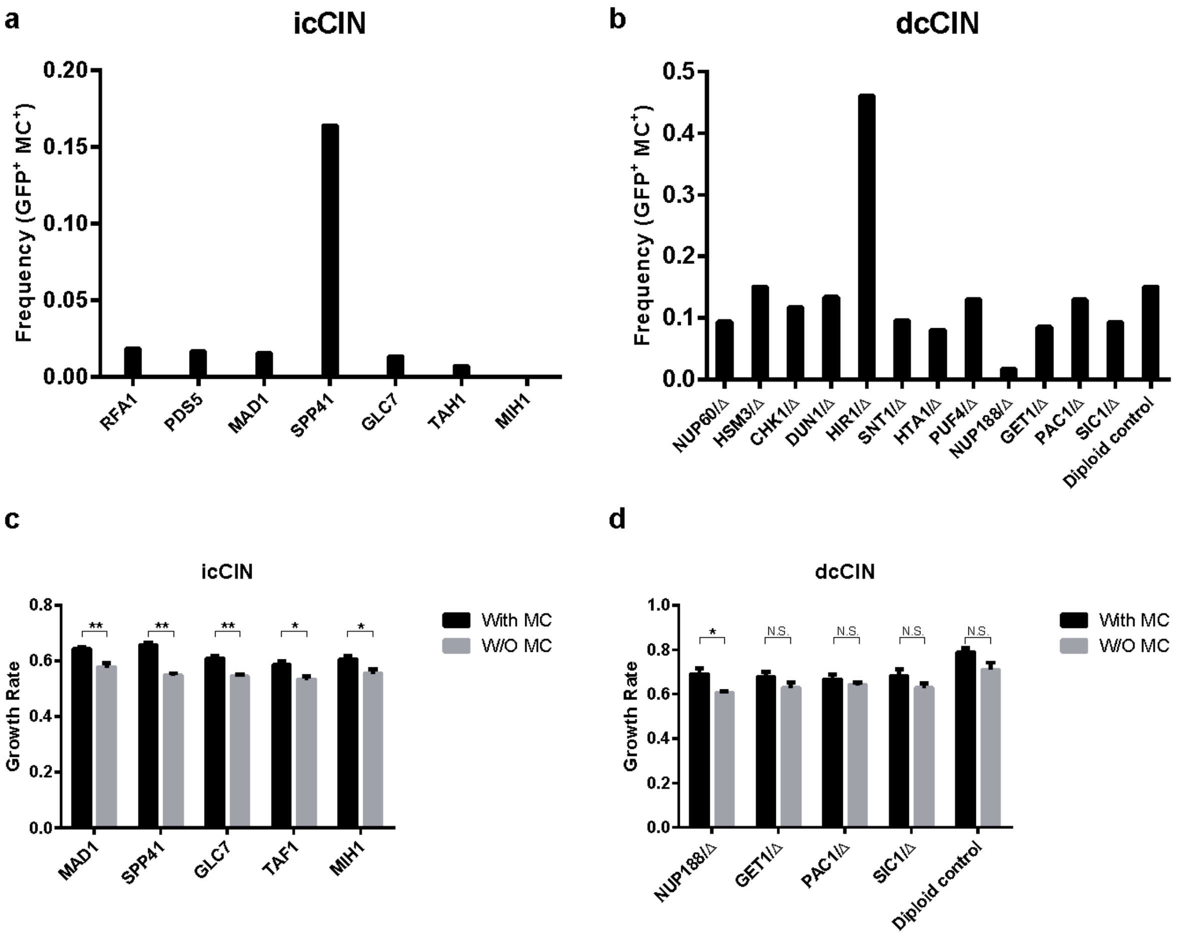

Figure S6 Additional validation results for the qCTF assay

a-b. Bar plots showing genetic changes other than MC loss that contribute to GFP^+ cell populations in selected hits from icCIN and dcCIN gene screens. Experiment was performed as described in Figure S1 b.

c-d. Bar plots comparing growth rates of MC^+ versus MC^- in some of the hit strains. Experiment was performed as described in Figure 1d. Data were shown as Mean ± SEM, n=8. P value was calculate from Mann Whitney. One asterisk, $p < 0.05$; two asterisks, $p < 0.01$; N.S.: non-significant.
