# Supplementary material for: Single-Cell Based Quantitative Assay of Chromosome Transmission Fidelity
Source: G3 (Bethesda). 2015 Mar 30;5(6):1043–56. doi: 10.1534/g3.115.017913 (PMC4478535; doi:10.1534/g3.115.017913)
Supplement: Supporting Information [file supp_g3.115.017913_FileS1.pdf]

File S1

Extended Materials and Methods

Formula for extrapolation of MC loss rate

We chose not to use the Luria-Delbrück method [1] for a practical reason: it requires multiple parallel cultures in order to calculate the mutation rate for one strain. Instead, we used a mutation accumulation model which allows the estimate of chromosome loss rate in one culture using two time point measurements. However, as Luria pointed out [2], to use this type of model, the population size must be big enough so that the probability that mutations occur in each generation is near unity. Our starting population has around 0.2 million cells, which is much larger than the population size used in fluctuation test (a few to a few hundred cells). Thus we could avoid random mutations to dominate the population. This can be seen in the tight CV (6%) when measuring 8 colonies of wild type cells. In addition, with this experimental design, the qCTF assay showed good agreement with the classic CTF assay (Figure 2a) which uses the half sectoring method to score chromosome loss rate.

1 Continuous time scale

Consider a continuous time model for the growth dynamics of the MC^+ cells that can transform back to MC^- state. Denote the number of MC^+ cells as $N^+(t)$ and MC^- cells as $N^-(t)$. The dynamics is described by a system of two linear ODE:

$$dN^+/dt = k_+N^+ - \alpha N^+, \quad N^+(0) = N_0^+, \tag{1}$$
$$dN^-/dt = k_-N^- + \alpha N^+, \quad N^-(0) = N_0^-, \tag{2}$$

where k_+, k_- are the growth rates of the corresponding strains, and α denotes the rate of chromosome loss. The solution of this system is given by

$$N^+(t) = N_0^+ e^{(k_+-\alpha)t}, \quad N^-(t) = N_0^- e^{k_-t} + \alpha N_0^+ \frac{e^{k_-t} - e^{(k_+-\alpha)t}}{\alpha + k_- - k_+}.$$

The fraction δ of MC^+ cells in the total population can be found as $\delta = N^+/(N^+ + N^-)$, so that

$$R = \frac{N^-}{N^+} = \frac{1-\delta}{\delta}.$$

The last ratio can be also written as

$$\frac{N^-}{N^+} = \frac{N_0^-}{N_0^+} e^{(\alpha+k_--k_+)t} + \alpha \frac{e^{(\alpha+k_--k_+)t} - 1}{\alpha + k_- - k_+}. \tag{3}$$

The last relation leads to direct computation of the loss rate α. Assuming in (3) that we can neglect unity compared to the exponential term we find an approximate relation

$$R = \left(R_0 + \frac{\alpha}{\alpha + k_- - k_+}\right) e^{(\alpha+k_--k_+)t}. \tag{4}$$

In case of zero chromosome loss rate $\alpha = 0$ we have

$$R = R_0 e^{(k_--k_+)t},$$

that shows that the MC^-/MC^+ population ratio in this case should grow exponentially with the rate $k_- - k_+$. In the particular case of equal growth rates ($k_+ = k_- = k$) for both strains we have

$$1 + R = (1 + R_0)e^{\alpha t},$$

leading to

$$e^{-\alpha t} = \frac{1 + R_0}{1 + R}.$$

Assuming that loss rate is small $\alpha \ll 1$ we rewrite the above relation

$$1 - \alpha t = \frac{1 + R_0}{1 + R}. \tag{5}$$

2 Discrete time scale: equal dounbling rates

Consider derivation of the corresponding formulas for the chromosome loss rate per cell division in the discrete case. Begin with the case of the equal growth rate (doubling time $T = (1/k)\ln 2$). Denote initial number (at time $t = 0$) of MC^+ and MC^- cells as N_0^+ and N_0^-, respectively. The loss rate $m < 1$ is the fraction of MC^+ cells that lose an additional chromosome and transform into MC^- cells. As both strains double with the same rate we find at the $t = nT$ the total number N_0 of cells in both strains is equal to $N_n = 2^n(N_0^+ + N_0^-)$. At each division the number of MC^+ cells doubles and decreases by fraction of m so that we find at n-th division $N_n^+ = 2(1-m)N_{n-1}^+$. Using this relation recursively we obtain

$$N_n^+ = [2(1-m)]^n N_0^+, \quad N_n^- = 2^n(N_0^+ + N_0^-) - N_n^+.$$

Find the ratio $R_n = N_n^-/N_n^+$ in the form

$$\begin{aligned} R_n &= \frac{N_n^-}{N_n^+} = \frac{2^n(N_0^+ + N_0^-) - [2(1-m)]^n N_0^+}{[2(1-m)]^n N_0^+} = \frac{N_0^+ + N_0^- - (1-m)^n N_0^+}{(1-m)^n N_0^+} \\ &= \frac{N_0^+ + R_0 N_0^+ - (1-m)^n N_0^+}{(1-m)^n N_0^+} = \frac{1 + R_0 - (1-m)^n}{(1-m)^n} = \frac{1 + R_0}{(1-m)^n} - 1. \end{aligned} \tag{6}$$

Rewrite the last relation as

$$(1-m)^n = \frac{1 + R_0}{1 + R_n}, \tag{7}$$

from which we find for small $m \ll 1$ an approximation

$$1 - mn = \frac{1 + R_0}{1 + R_n}. \tag{8}$$

Recall the formula (5) write it for $t = nT$ when $R = R_n$

$$1 - \alpha nT = \frac{1 + R_0}{1 + R_n},$$

comparing it with (8) we find for a relation between the loss rates in continuous and discrete cases $m = \alpha T = (\alpha/k)\ln 2$. Using (7) we obtain the formula for loss rate per doubling as

$$m = 1 - \left(\frac{1 + R_0}{1 + R_n}\right)^{1/n}, \quad n = \log_2 \frac{N_n^+}{N_0^+}. \tag{9}$$

Using smallness of m we have from (8)

$$m = \frac{R_n - R_0}{n(1 + R_n)}, \quad n = \log_2 \frac{N_n^+}{N_0^+}. \tag{10}$$

3 Discrete time scale: unequal dounbling rates

Consider the general case of unequal doubling times $T_- = (1/k_-)\ln 2$ for MC$^-$ and $T_+ > T_-$ for MC$^+$ cells and divisions are numbered in MC$^+$ scale, i.e., at times $t = nT_+$. At $t = T_+ = (1/k_+)\ln 2$ we have for MC$^+$ strain $N_1^+ = 2(1-m)N_0^+$ cells while for MC$^-$ strain $N_1^- = 2^{T_-/T_+}N_0^- + 2mN_0^+$. Introduce the ratio $\beta = T_-/T_+$, of the doubling times. We write for the first three divisions

n	N_n^-	N_n^+
0	N_0^-	N_0^+
1	$2^\beta N_0^- + 2mN_0^+$	$2(1-m)N_0^+$
2	$2^\beta(2^\beta N_0^- + 2mN_0^+) + 4m(1-m)N_0^+$	$[2(1-m)]^2 N_0^+$
3	$2^\beta(2^\beta(2^\beta N_0^- + 2mN_0^+) + 4m(1-m)N_0^+) + 8m(1-m)^2 N_0^+$	$[2(1-m)]^3 N_0^+$

Consider the expression for N_2^-

$$N_2^- = (2^\beta)^2 N_0^- + 2^\beta 2mN_0^+ + 4m(1-m)N_0^+ = (2^\beta)^2 N_0^- + 2mN_0^+[2^\beta + 2(1-m)]$$

$$= (2^\beta)^2 N_0^- + 2mN_0^+ 2^\beta(1+q),$$

where $q = 2^{1-\beta}(1-m)$. Expanding the expression for N_3^- we have

$$N_3^- = (2^\beta)^3 N_0^- + (2^\beta)^2 2mN_0^+ + 2^\beta 2^2 m(1-m)N_0^+ + 2^3 m(1-m)^2 N_0^+$$

$$= (2^\beta)^3 N_0^- + 2mN_0^+(2^\beta)^2(1+q+q^2),$$

and we find

$$N_3^- = 2^{3\beta}\left[N_0^- + 2^{1-\beta}mA_0(1+q+q^2)\right] = 2^{3\beta}\left[N_0^- + 2^{1-\beta}mA_0\frac{1-q^3}{1-q}\right].$$

It is easy to see the pattern in the expressions for N_n^- and N_n^+

$$N_n^- = 2^{n\beta}\left[N_0^- + 2^{1-\beta}mA_0\frac{1-q^n}{1-q}\right], \quad N_n^+ = [2(1-m)]^n N_0^+.$$

Using these general expressions in definition of the ratio R_n we obtain

$$R_n = \frac{N_n^-}{N_n^+} = \frac{2^{n\beta}[N_0^- + 2^{1-\beta}mA_0\frac{1-q^n}{1-q}]}{[2(1-m)]^n N_0^+} = \left[\frac{2^{\beta-1}}{1-m}\right]^n \left[\frac{N_0^-}{N_0^+} + 2^{1-\beta}m\frac{1-q^n}{1-q}\right].$$

Using the definitions of R_0 and q we rewrite the above relation

$$q^n R_n = R_0 + 2^{1-\beta}m\frac{1-q^n}{1-q} = R_0 + \left(2^{1-\beta} - q\right)\frac{1-q^n}{1-q}. \quad (11)$$

Here n is the number of divisions of the MC$^+$ cells found for time t as

$$n = \frac{k_+ t}{\ln 2}.$$

Solving the equation (11) for q given values of R_0, R_n, and $\beta = T_-/T_+$ we find the loss rate m of the MC$^+$ cells per doubling time of this strain

$$m = 1 - 2^{\beta-1}\,q = 1 - 2^{(T_- - T_+)/T_+}\,q. \quad (12)$$

For the equal doubling times $T_+ = T_- = T$ we have $\beta = 1$ and the relation (11) reduces to (9).

Assuming $\beta \approx 1$ we set $\beta = 1 - \gamma$, where $\gamma \ll 1$. Thus we have

$$m + 2^{-\gamma} q = m + (1 - \gamma \ln 2) q = 1. \tag{13}$$

It is clear that $q \approx 1$, i.e., $q = 1 - \delta$ with $\delta \ll 1$. Using the last approximation transform the relation (11) into

$$2(R_n - R_0) + 2n(1 - 2^\gamma) - n[n(1 - 2^\gamma) + (1 + 2^\gamma) - 2R_n]\delta = 0,$$

from which we find the value of δ

$$\delta = 2\frac{R_n - R_0 + n(1 - 2^\gamma)}{n[n(1 - 2^\gamma) + (1 + 2^\gamma) - 2R_n]}. \tag{14}$$

From (13) it follows that

$$m = 1 - (1 - \delta)2^{-\gamma} = \frac{R_n - R_0}{n(1 + R_n)} \left[1 + \gamma \ln 2 \frac{n(3R_n - R_0) - (R_n - R_0) + 2(n-1)R_n^2 + 2R_n R_0}{2(1 + R_n)(R_n - R_0)}\right].$$

Compare this result with (10) we see that in case when the ratio of the doubling times of the two strains is close to unity the rate of mutation m is given by the formula (10) with a small correction proportional to the value of $\gamma = 1 - T_-/T_+$. As we have $R_0 < R_n \ll 1$ the correction reads $3\gamma n/2 \approx \gamma n$, and when $\gamma n \sim 1$, this correction cannot be neglected.

References

[1] Luria, S. E., and M. Delbrück, Mutations of Bacteria from Virus Sensitivity to Virus Resistance. *Genetics* **28** 491-511, 1943.

[2] Luria, S. E., 1951 The Frequency Distribution of Spontaneous Bacteriophage Mutants as Evidence for the Exponential Rate of Phage Reproduction. *Cold Spring Harbor Symposia on Quantitative Biology* **16** 463-470, 1951.
